# High-Molecular-Weight Fractions of Spruce and Eucalyptus Lignin as a Perspective Nanoparticle-Based Platform for a Therapy Delivery in Liver Cancer

**DOI:** 10.3389/fbioe.2021.817768

**Published:** 2022-02-07

**Authors:** Ievgen V. Pylypchuk, Huizhen Suo, Chanakarn Chucheepchuenkamol, Nils Jedicke, Pär A. Lindén, Mikael E. Lindström, Michael P. Manns, Olena Sevastyanova, Tetyana Yevsa

**Affiliations:** ^1^ Division of Wood Chemistry and Pulp Technology, Department of Fiber and Polymer Technology, School of Chemistry, Biotechnology and Health, KTH Royal Institute of Technology, Stockholm, Sweden; ^2^ Department of Materials and Environmental Chemistry, Stockholm University, Stockholm, Sweden; ^3^ Department of Gastroenterology, Hepatology and Endocrinology, Hannover Medical School, Hannover, Germany; ^4^ Department of Science Service, Ministry of Higher Education, Science, Research and Innovation, Ratchathewi, Thailand; ^5^ Wallenberg Wood Science Center, Department of Fiber and Polymer Technology, School of Chemistry, Biotechnology and Health, KTH Royal Institute of Technology, Stockholm, Sweden

**Keywords:** lignin nanoparticles (LNPs), cancer treatment, eucalyptus lignin, spruce lignin, primary liver cancer (PLC), hepatocellular carcinoma (HCC), cholangiocarcinoma (CCA), apoptosis

## Abstract

The natural polymer, lignin, possesses unique biodegradable and biocompatible properties, making it highly attractive for the generation of nanoparticles for targeted cancer therapy. In this study, we investigated spruce and eucalyptus lignin nanoparticles (designated as S-and E-LNPs, respectively). Both LNP types were generated from high-molecular-weight (M_*w*_) kraft lignin obtained as insoluble residues after a five-step solvent fractionation approach, which included ethyl acetate, ethanol, methanol, and acetone. The resulting S-and E-LNPs ranged in size from 16 to 60 nm with uniform spherical shape regardless of the type of lignin. The preparation of LNPs from an acetone-insoluble lignin fraction is attractive because of the use of high-M_*w*_ lignin that is otherwise not suitable for most polymeric applications, its potential scalability, and the consistent size of the LNPs, which was independent of increased lignin concentrations. Due to the potential of LNPs to serve as delivery platforms in liver cancer treatment, we tested, for the first time, the efficacy of newly generated E-LNPs and S-LNPs in two types of primary liver cancer, hepatocellular carcinoma (HCC) and cholangiocarcinoma (CCA), *in vitro*. Both S-LNPs and E-LNPs inhibited the proliferation of HCC cells in a dose-dependent manner and did not affect CCA cell line growth. The inhibitory effect toward HCC was more pronounced in the E-LNP-treated group and was comparable to the standard therapy, sorafenib. Also, E-LNPs induced late apoptosis and necroptosis while inhibiting the HCC cell line. This study demonstrated that an elevated number of carbohydrates on the surface of the LNPs, as shown by NMR, seem to play an important role in mediating the interaction between LNPs and eukaryotic cells. The latter effect was most pronounced in E-LNPs. The novel S- and E-LNPs generated in this work are promising materials for biomedicine with advantageous properties such as small particle size and tailored surface functionality, making them an attractive and potentially biodegradable delivery tool for combination therapy in liver cancer, which still has to be verified *in vivo* using HCC and CCA models.

**GRAPHICAL ABSTRACT:**
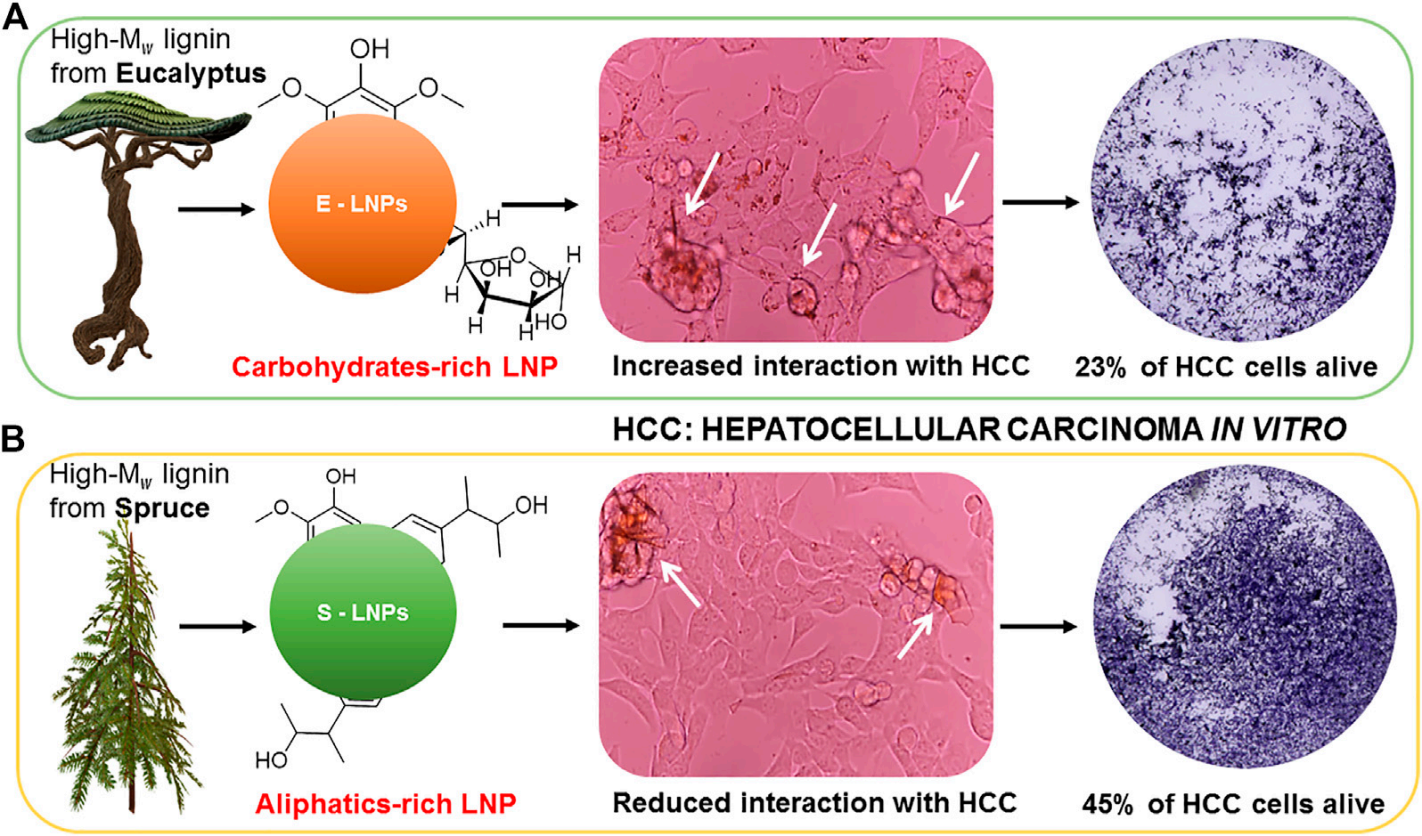
The differences in the surface chemistry of the LNPs influence their interaction with cancerous cells and might mediate increased biotoxicity of the E-LNPs. Shown are **(A)** the elevated content of the *carbohydrates* on the E-LNP surface, favoring their interaction with cells; **(B)** the elevated abundance of the *hydroxypropenyl* units on the surface of S-LNP seems to hamper their ability to interact with cancerous cells.

## Introduction

Primary liver cancer represents the third leading cause of cancer-related death worldwide (Craig et al., 2020). Primary liver carcinomas with either hepatocytic or cholangiocytic differentiation are referred to hepatocellular carcinoma (HCC) and cholangiocarcinoma (CCA), respectively (Brunt et al., 2018). HCC represents the most common form of primary liver cancer and typically develops on a background of chronic liver disease, with Hepatitis B (HBV) and Hepatitis C (HCV) virus infection, alcohol abuse, and nonalcoholic fatty liver disease being the major etiologies (Craig et al., 2020). Sorafenib has been used as standard therapy for HCC since 2007 (Llovet et al., 2008), and recently immunotherapy was introduced as first-line therapy in HCC patients (Nenu et al., 2018; Johnston and Khakoo, 2019; Xu et al., 2019). CCA is the second most common primary hepatic malignancy after HCC (Nakeeb et al., 1996; DeOliveira et al., 2007; Banales et al., 2016; Banales et al., 2020) and includes a cluster of highly heterogeneous biliary malignant tumors, arising at any point of the biliary tree (Banales et al., 2020). Case–control studies and meta-analyses defined several risk factors to CCA, including HBV and HCV infections, biliary tract diseases like cholangitis, choledochal cyst, cholelithiasis, cirrhosis, and environmental toxins (Clements et al., 2020). Current standard first-line systemic therapy for patients with advanced CCA comprises a combination of gemcitabine and cisplatin; however, immunotherapy has started to play a more important role (Hochnadel et al., 2017; Guo and Shen, 2020; Walcher et al., 2020).

Yet, for both HCC and CCA the development of new therapies is in high need. Special importance is given to combination therapies. The development of platforms capable of targeted delivery of therapeutics locally to the liver tumors are of high clinical relevance, as recently reviewed (Hochnadel et al., 2017; Walcher et al., 2020).

Nanoparticles (NPs), nanoliposomes, and other nanomedicine objects are advantageous in targeting malignant solid tumors because they have small sizes (<1,000 nm) and have unique structures that can amplify the anticancer activity of conventional drugs (Tang et al., 2007; Lee et al., 2008; Towata et al., 2010; Helio et al., 2011; Chung et al., 2012; Hwang et al., 2013; Kwak et al., 2015). However, current nanotechnology in liver cancer is represented mainly by NPs of inorganic origin (calcium carbonate (CaCO_3_), iron oxide (Fe_3_O_4_), gold (Au), silver, platinum, and others) in both cancer diagnosis and treatment (Bhattacharya and Mukherjee, 2008; Hanafy et al., 2016; Hanafy, 2017; Moghadam, 2017; Taghizadeh et al., 2019; Figueiredo et al., 2021) with limitations in terms of biodegradability and biocompatibility.

In this view, lignin, as the most abundant natural aromatic polymer on Earth, has been shown to be an efficient platform for drug loading through the preparation of lignin nanoparticles (LNPs) (Sipponen et al., 2019; Osterberg et al., 2020; Figueiredo et al., 2021). LNPs, often referred to as colloidal lignin particles (CLPs) (Sipponen et al., 2018), have unique and different features compared to a large number of other materials at the same source (De Jong and Borm, 2008; Gao and Fatehi, 2019). For instance, they have a high surface area and a much larger surface-to-mass ratio than other types of particles that are prepared from natural polymers (De Jong and Borm, 2008). Additionally, it is worth mentioning that the utilization of lignin contributes to United Nations (UN) sustainable development goals, such as CO_2_ reduction and the usage of non-fossil resources for the development of the bio-based circular economy (Korhonen et al., 2018). As a natural polyphenol material, lignin is biodegradable and has shown high antioxidant (Pan et al., 2006) and antibacterial properties (Kaur et al., 2017). Moreover, LNPs prepared from kraft lignin have been investigated as a treatment of prostate, mammary, lung, and breast cancer, in works by Figueiredo et al. (2017a), Figueiredo et al. (2017b), Figueiredo et al. (2019), and Figueiredo et al. (2020), Siddiqui et al. (2020), and others. Furthermore, eucalyptus kraft lignin has been recently shown to possess inhibitory properties in mouse hepatoma and melanoma (Gordobil et al., 2019) and some components extracted from grass lignin were active against breast cancer (Zhen et al., 2020). LNPs loaded with active substances have been hypothesized to be efficient in colon cancer suppression (Siddiqui et al., 2018).

Taking into consideration the strong potential of LNPs to serve as drug delivery systems, in the present work we aimed to study the efficacy of LNPs originating from the high-molecular weight (M_*w*_) fractions of eucalyptus (E-LNPs) and spruce (S-LNPs) in aggressive liver cancer. The novel S- and E-LPNs generated in this study not only showed efficacy toward liver cancer but additionally showed a set of advantageous parameters that would be further useful in biomedicine.

## Experimental Procedures

### Materials

Kraft lignin from hardwood (*Rose Gum Eucalyptus grandis*) and softwood (Norway spruce, *Picea abies*) was isolated from corresponding black liquors according to the LignoBoost process (Tomani, 2010). Ultrapure water (18.2 MΩ-cm) was obtained from the Milli-Q system. Tetrahydrofuran (THF), DMSO (dimethyl sulfoxide, anhydrous, ≥99.9%), deuterated DMSO (DMSO-d6), and deuterated water (D_2_O) were purchased from Sigma-Aldrich (Stockholm, Sweden). Acetone (GPR RECTAPUR^®^, ≥99.5%) was purchased from VWR International AB (Stockholm, Sweden), and 0.45-µm membrane filters (Fisherbrand, PTFE, Fisher Scientific, Hampton, NH, United States) were used for the isolation procedure.

### Lignin Fractionation

For lignin fractionation, we used a solvent fractionation approach of kraft lignin described elsewhere (Duval et al., 2016). The solvent order used for the fractionation was as follows: ethyl acetate (EtOAc) (N1 solvent), ethanol (EtOH) (N2 solvent), methanol (MeOH) (N3 solvent), and acetone (N4 solvent). The full characteristics of the lignin fractions have been reported previously (Gioia et al., 2020). Briefly, 20 g of lignin powder was extracted with 200 ml of solvent N1 for 2 h at room temperature. The soluble fraction was decanted, the solvent was evaporated, and lignin materials were freeze-dried for 3 days. The remained insoluble fraction was suspended in the next solvent N2, and the next extraction step was performed similarly. The procedure was repeated with all remaining solvents described above. The residual lignin fraction, not soluble in abovementioned organic solvents, was numbered as a fraction N5. The fraction N5 was used in this work to synthesize the LNPs.

### Characterization of Lignin

#### Nuclear Magnetic Resonance (^31^P NMR and 2D NMR)

The numbers of aliphatic hydroxyls (Aliph-OH), total phenolic hydroxyls (Ph-OH), and guaiacyl and syringyl units in the initial lignin samples were measured and calculated using quantitative ^31^P NMR (Argyropoulos, 1994; Granata and Argyropoulos, 1995). For this, the lignin sample (about 30 mg) was dissolved in 100 µl of DMF and 100 µl of pyridine. Endo-N-hydroxy-5-norbornene-2,3-dicarboximide (e-HNDI) (Sigma-Aldrich, 40 mg/ml) and chromium(III) acetylacetonate (Aldrich, St. Louis, MO, United States, 5 mg/ml) were used as an internal standard and relaxation reagent, respectively. 2-Chloro-4,4,5,5-tetramethyl-1,3,2-dioxaphospholane was used as a phosphorylating reagent. The derivatized sample was dissolved in deuterochloroform (CDCl_3_) prior to analysis. The ^31^P NMR experiment was performed with a 90° pulse angle, inverse-gated proton decoupling, and a delay time of 10 s. For analysis, 256 scans with a time delay of 5 s were collected for a total runtime of 29 min.

To estimate the number of ß-O-4 bonds, 2D NMR analysis was performed as previously described (Giummarella et al., 2020; Jawerth et al., 2020). For this, approximately 80 mg of lignin solution was dissolved in deuterated DMSO-d6. Distortionless enhancement by polarization transfer (DEPT)-edited heteronuclear single quantum correlation (HSQC) and HSQC experiments were carried out with the Bruker pulse programs “hsqcedetgp” and “hsqcetgpsi,” respectively. The spectra were acquired with the following parameters: an acquisition time of 0.06 s, a relaxation delay 1.5 s, and 120 scans using 1,024 × 256 increments, with an additional 16 dummy scans. The F2 direction spectral window was 16 ppm, and the F1 direction was 166 ppm. The analysis lasted 14 h. Data were processed in MestreNova (version 9.0.0, Mestrelab Research, Santiago de Compostela, Spain) with 1,024 × 1,024 data points using a 90°-shifted square sine-bell apodization window.

#### M_*w*_ Determination

Determination of M_*w*_ of the lignin samples was performed after the acetobromination reaction by size-exclusion chromatography (SEC), according to the protocols reported by Guerra et al. (2006) and Tagami et al. (2019). A Waters instrument system (Waters Sverige AB, Sollentuna, Sweden) consisting of a 515 high-performance liquid chromatography (HPLC) pump, 2707 autosampler, and 2998 photodiode array detector (operated at 254 and 280 nm) was used. Lignin samples (about 5 mg) were stirred for 2 h at room temperature in 900 μl of glacial acetic acid and 100 μl of acetyl bromide. Thereafter, a mixture of glacial acetic acid and acetyl bromide was removed under nitrogen (N_2_). The residue was dissolved in 1 ml of HPLC-grade THF (Sigma, Sweden), and the resulting solution was filtered through a 5-μm syringe filter. HPLC-grade THF was used as a mobile phase. The flow rate of the mobile phase was 0.3 ml/min. The Waters Ultrastyragel HR4, HR2, and HR0.5 4.6 × 300 mm columns connected in series and operated at 35°C were used for the separation. A sample volume was 20 μl, and the ultraviolet (UV) signal was recorded at 254 and 280 nm. Calibration was performed at 254 nm using polystyrene standards with nominal M_*W*_ values ranging from 480 to 176,000 Da. The final analysis was performed using the intensity of the UV signal at 280 nm using Waters Empower 3 build 3471 software.

#### Analytical Pyrolysis (Py-GC/MS/FID)

Analytical pyrolysis-gas chromatography/mass spectrometry/flame ionization detector (Py-GC/MS/FID) was performed according to Ponomarenko et al. (2015) using a Frontier Lab (Fukushima, Japan) Micro Double-Shot Pyrolyzer Py-2020iD. Approximately 1–2 mg of dry sample was used for analysis. The oven program was as follows: held at 60°C for 1 min, ramped at 6°C/min^−1^–270°C, and held at 270°C for 10 min. The individual compounds were identified based on the GC/MS data in the NIST 147.LI13 MS library (Frontier Lab, Fukushima, Japan). The relative areas of the peaks of individual compounds were calculated from the GC/FID data using Shimadzu software. The areas of the relevant peaks were summed and normalized to 100%; the data for five repetitive pyrolysis experiments were averaged (Tagami et al., 2019).

#### Preparation of Lignin Nanoparticles

The LNPs from fraction N5 of eucalyptus lignin were designated as E-LNPs (eucalyptus NPs), and the LNPs from spruce lignin fraction N5 were designated as S-LNPs (spruce NPs) in this work. A dialysis bag (Sigma-Aldrich) was used to synthesize the LNPs by the solvent exchange method (DeOliveira et al., 2007). The M_*w*_ cutoff of the dialysis bag was 1,000 Da. Spruce or eucalyptus acetone-insoluble fractions were dissolved in DMSO/Milli-Q water solution with the ratio 9:1 (v/v) and a concentration of 10 mg/ml. The resulting lignin solution was filtered using a 0.45-μm filter. After filtration, 10 ml of the lignin solution with a concentration of 1–6 mg/ml was prepared by dilution with a corresponding solvent mixture from the lignin stock solution. To obtain the LNPs, the resulting lignin solution was placed into the dialysis bag and soaked in Milli-Q water for 36 h. The experiment was performed at room temperature. The volume of the lignin solution before and after placing it into the dialysis bag was measured to estimate the final lignin concentration in the resulting LNP solution.

### Characterization of Lignin Nanoparticles

#### Nuclear Magnetic Resonance (^1^H NMR)

The surface chemistry of LNPs in aqueous suspension was elucidated using an ^1^H NMR approach as described in our previous work (Pylypchuk et al., 2020). After centrifugation, ca. 0.75 mg of LNPs was dispersed in a mix of 0.2 ml of D_2_O (Sigma-Aldrich, Sweden) and 0.5 ml of H_2_O and analyzed on a Bruker DMX instrument (Bruker Corporation, Billerica, MA, United States) equipped with a 5-mm Bruker Double Resonance Broadband Probe (BBI) probe (Bruker Corporation, Billerica, MA, United States). Optimal 90° pulse lengths were obtained for each sample, after which the same pulse program as described in (Pylypchuk et al., 2020) was applied. 512 scans with 20,030 FID data points were obtained per sample, with an acquisition time of 2.5 s and a relaxation delay time of 4.5 s. The data were processed in TopSpin (version 1.3, patch level 10, Bruker BioSpin) with 32,768 data points using a 0.3-Hz exponential multiplication apodization window before being transferred to MestreNova (version 9.0.0, Mestrelab Research), where the final processing was performed.

#### Dynamic Light Scattering

The zeta ζ-potential, average size, size distribution, and polydispersity indices (PDI) of the synthesized LNPs were determined using a Zetasizer Nano ZS instrument (Malvern Panalytical, Malvern, United Kingdom) at 25°C and at 37°C.

#### Transmission Electron Microscopy

For TEM, the samples were prepared as follows: 5 µl of lignin LNP suspension (0.2 mg/ml) was drop-cast onto a 200-mesh copper grid (Ted Pella Inc., Redding, CA, United States; prod no. 01800-F) and dried on air for 30 min. To perform TEM studies, a Hitachi HT7700 series instrument (Hitachi, Japan) was used with an accelerating voltage of 100.0 kV and an emission current of 80.0 µA.

#### Scanning Electron Microscopy

The LNP solution was drop-cast on a silicon wafer for 30 min and sputter-coated with a 2-nm layer of Pt–Pd. An S-4800 microscope was used (S-4800 Hitachi, Japan) in SEM studies.

#### Establishment of HCC and CCA Cell Lines

HCC and CCA cell lines were isolated from murine primary liver tumors. HCC was induced using the intrahepatic overexpression of oncogenic *NRAS* ^*G12V*^ stably delivered into the liver of mice in which tumor-suppressor *Arf* is disabled (p19^Arf−/−^ mice) *via* hydrodynamic tail vein injection, as previously described (Carlson et al., 2005; Kang et al., 2011). CCA was induced using a stable intrahepatic integration of mixture with transposons encoding *KRAS* ^*G12V*^ and *Akt2* oncogenes, as well as short hairpins against p53 (*shp53*), using the electroporation technique as established (Gürlevik et al., 2013; Gürlevik et al., 2016). Obtained HCC and CCA cell lines were cultured in Dulbecco’s Modified Eagle’s Medium (DMEM; Gibco, Grand Island, NY, United States) supplemented with 10% fetal bovine serum (FBS; Gibco, United States), 5% penicillin/streptomycin/glutamine (Gibco, United States), and 5% Minimum Essential Medium Non-Essential Amino Acids (MEM NEAA, Gibco, United States). The cells were incubated at 37°C in a humidified incubator supplied with 5% carbon dioxide.

#### Treatment of HCC and CCA Cell Lines With Lignin Nanoparticles

HCC and CCA cells were seeded in 96-well plates (Corning, Inc., Tewksbury, MA, United States) at a density of 1 × 10^4^ cells per well. Two LNP types—spruce (S-LNPs) or eucalyptus (E-LNPs)—as well as the standard therapeutics (sorafenib for HCC and gemcitabine for CCA) were added to the wells 16 h later in triplicates. Several concentrations of the therapeutics were tested in parallel. The concentrations of LNPs comprised 0.5, 1, 3, 4, 8, and 13.8 µM or 0.03, 0.06, 0.19, 0.21, 0.50, and 0.86 μg/ml, corresponding to 4.77 × 10^11^, 9.55 × 10^11^, 2.86 × 10^12^, 3.29 × 10^12^, 7.64 × 10^12^, and 1.32 × 10^13^ NPs/ml and Supplementary Table S1). Thereafter, different readouts were conducted at 24 and 48 h of post-incubation with LNPs and therapeutics.

#### Crystal Violet Staining Assay

Crystal violet staining assay (CVSA) was performed as reported (Rudalska et al., 2014; Śliwka et al., 2016). Briefly, cells were washed with 100 µl 1× PBS and fixed in 100 µl of 4% paraformaldehyde for 5 min. Thereafter, cells were stained with 100 µl of 0.5% crystal violet (Sigma-Aldrich Corp., St. Louis, MO, United States) in 30% EtOH for 20–30 min at room temperature. Finally, wells were washed in tap water and dried overnight. The pictures were captured using an ImmunoSpot® S6 Ultimate Analyzer (Cellular Technology Limited, Shaker Heights, OH, United States). The obtained images were analyzed using ImageJ software (https://imagej.nih.gov/ij/index.html).

#### Cell proliferation assay/Cell Counting Kit-8

Cell Counting Kit-8 (CCK-8) assay (Sigma-Aldrich, United States) was used to examine cell division and was conducted using a manufacturer protocol. Cell proliferation was checked after 24 and 48 h of treatment with LNPs. On each day, 10 µl of CCK-8 solution was added to each well. After 2 h of incubation, the absorbance of each well was measured using an Infinite 200 PRO Nano Quant Tecan Microplate Readers and analyzed using i-control™ software (Zhou et al., 2018) (Tecan, CH-8708, Mannedorf, Switzerland).

#### Analysis of Early- and Late Apoptosis, and Necroptosis, Using Flow Cytometry (FACS)

After the treatment with LNPs, cells were harvested, washed with ice-cold PBS, and resuspended in 100 µl of Annexin V binding buffer (10 mM HEPES [pH 7.4], 140 mM NaCl, 2.5 mM CaCl_2_), provided by a manufacturer (BioLegend^®^, San Diego, CA, United States), as described (Yang et al., 2016). Next, cells were stained with 5 µl of Annexin V-PE (BioLegend^®^, United States) and 5 μg/ml of 7-AAD (BioLegend^®^, United States) in 100 µl of Annexin V binding buffer at 4°C. After 20 min, 400 µl of binding buffer was added to each tube. The samples were pooled within each group and analyzed using a flow cytometer (BD™ LSR II, San Jose, CA, United States). FACS analysis was performed using the FlowJo 9.9.6 software (BD™, Franklin Lakes, NJ, United States).

### Statistical Analysis

The unpaired Student’s *t*-test was used for all statistical analyses to calculate significant differences among experimental and control groups. The tests were generally performed in duplicates or triplicates. If not stated otherwise, data are shown as mean ± standard error of the mean (S.E.M.) with *p* < 0.05 considered statistically significant. Significance levels were depicted as *p < 0.05, **p < 0.01, ***p < 0.001, and ****p < 0.0001. Data on particle size and ζ-potential are shown as mean ± standard deviation (SD), which were extracted from the original software.

## Results and Discussion

### Solvent Fractionation Approach Gave Rise to High-M_*w*_- and Carbohydrate-Rich Lignins

Lignin is a heterogeneous substance, and it is not always possible to establish a relationship between the structure and properties of materials derived from lignin (Gioia et al., 2020). To cope with lignin heterogeneity, the solvent fractionation approach was proposed (Duval et al., 2016) and has been successfully applied to tailor the properties of lignin and its materials on the molecular level (Gioia et al., 2018; Giummarella et al., 2020). Nevertheless, the so-called insoluble fractions remaining after solvent fractionation have yet to be sufficiently studied and are therefore underutilized as materials because of their poor solubility in most organic solvents (Gioia et al., 2018; Giummarella et al., 2020). However, the high-M_*w*_ and chemical structure of acetone-insoluble fractions makes it the closest in structure to native lignin in wood, bearing more β-O-4 bonds (Olsen et al., 2019). Moreover, a relatively high carbohydrate content makes acetone-insoluble fractions potentially more biocompatible.

**FIGURE 1:**
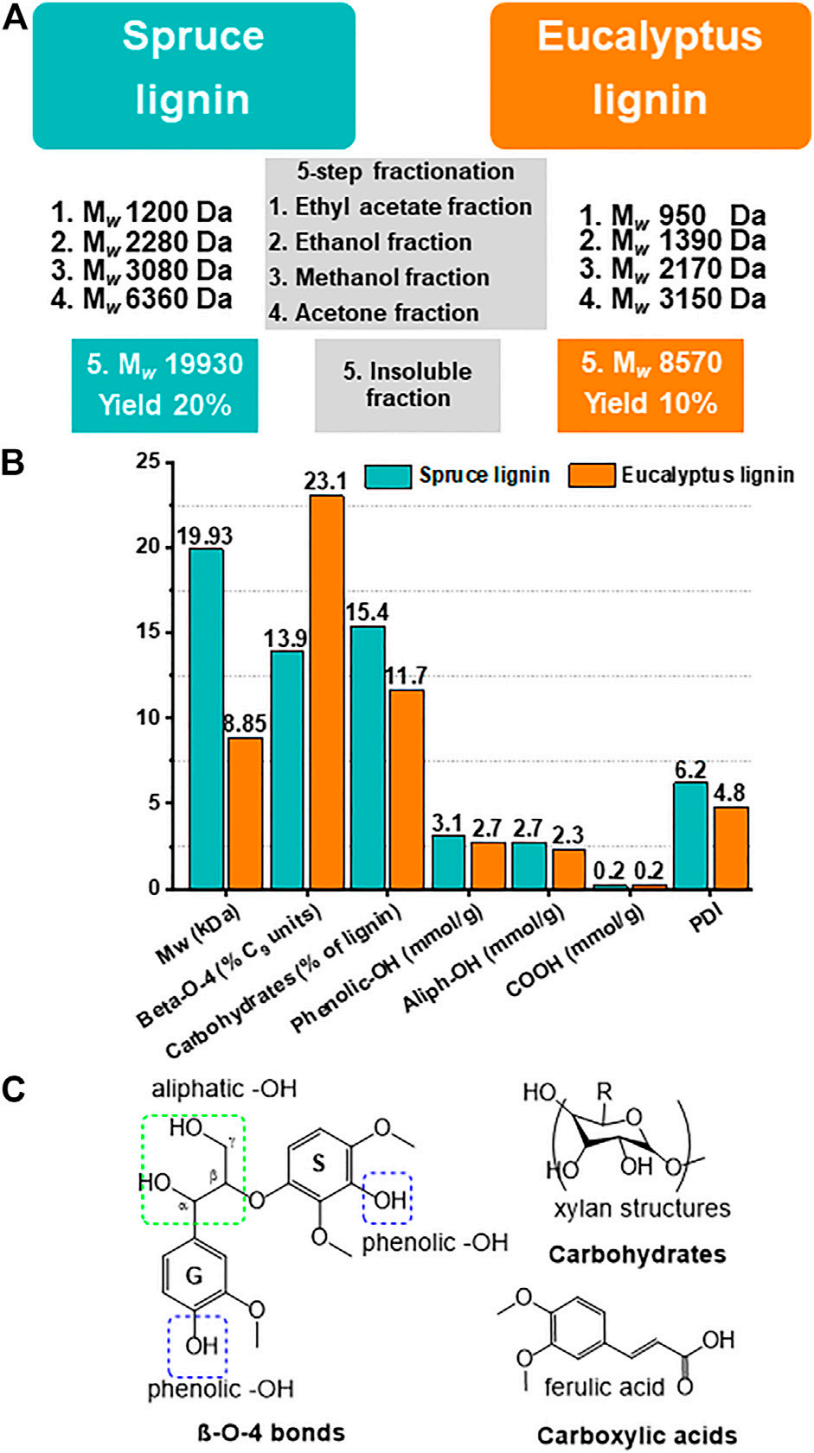
The highest M_*w*_ lignin fractions were used in this study to synthesize the LNPs. **(A–C)** The properties of kraft lignins and isolated fractions thereof used in this work. **(A)** Scheme of kraft lignin separation by M_*w*_ using sequential 5-step solvent fractionation. The residual fraction with the highest M_*w*_ obtained at the last fractionation step (insoluble fraction N5) was used for LNP preparation. **(B)** Average M_*w*_ and polydispersity indices (PDI) of the residual fraction of spruce and eucalyptus lignin, as determined by size-exclusion chromatography (SEC). The functional group content, determined by ^31^P NMR, demonstrating that both lignin types have relatively high aliphatic-hydroxyls (Aliph-OH) and low aromatic-OH content, as was determined (Tagami et al., 2019). **(C)** Depicted are the most common chemical groups like aliphatic-OH, aromatic-OH, carbohydrates and carboxylic acids, and linkages (ß-O-4) in the natural lignin molecule.

In the present work, the high-M_*w*_ lignin fractions were obtained by separating lignin using a 5-step multi-solvent fractionation approach (Figure 1A). This approach leads to higher yields and more efficient separation of lignin molecules by M_*w*_, as has been shown in the work of Tagami et al. (2019). The structure and M_*w*_ of the obtained acetone-insoluble fractions have been investigated using ^31^P NMR and analytical pyrolysis (Py-GC-MS-FID), as reported previously (Tagami et al., 2019). The acetone-insoluble lignin fraction obtained from spruce (softwood) demonstrated higher M_*w*_ than the one obtained from eucalyptus (hardwood), as presented in Figure 1B. Moreover, initial spruce lignin (not the S-LNPs) contained a higher amount of carbohydrates (15.4% for spruce and 11.7% for eucalyptus, respectively) (Figure 1B). Except for the polydispersity indices (PDI), all other tested parameters remained similar between these lignins (Figure 1B). Also, the high-M_*w*_ fractions of spruce and eucalyptus lignins contained a high amount of aliphatic side chains, probably due to a higher number of interunit linkages (Figure 1C), where the aromatic rings were not connected directly to each other, resulting in lower aromatic density as it was shown by Olsen et al. (2019).

### The LNPs Resulting from High-M_*w*_ Lignin Fractions Demonstrated a Size Below 100 nm which was Independent of Initial Pre-Dialysis Lignin Concentrations

**FIGURE 2:**
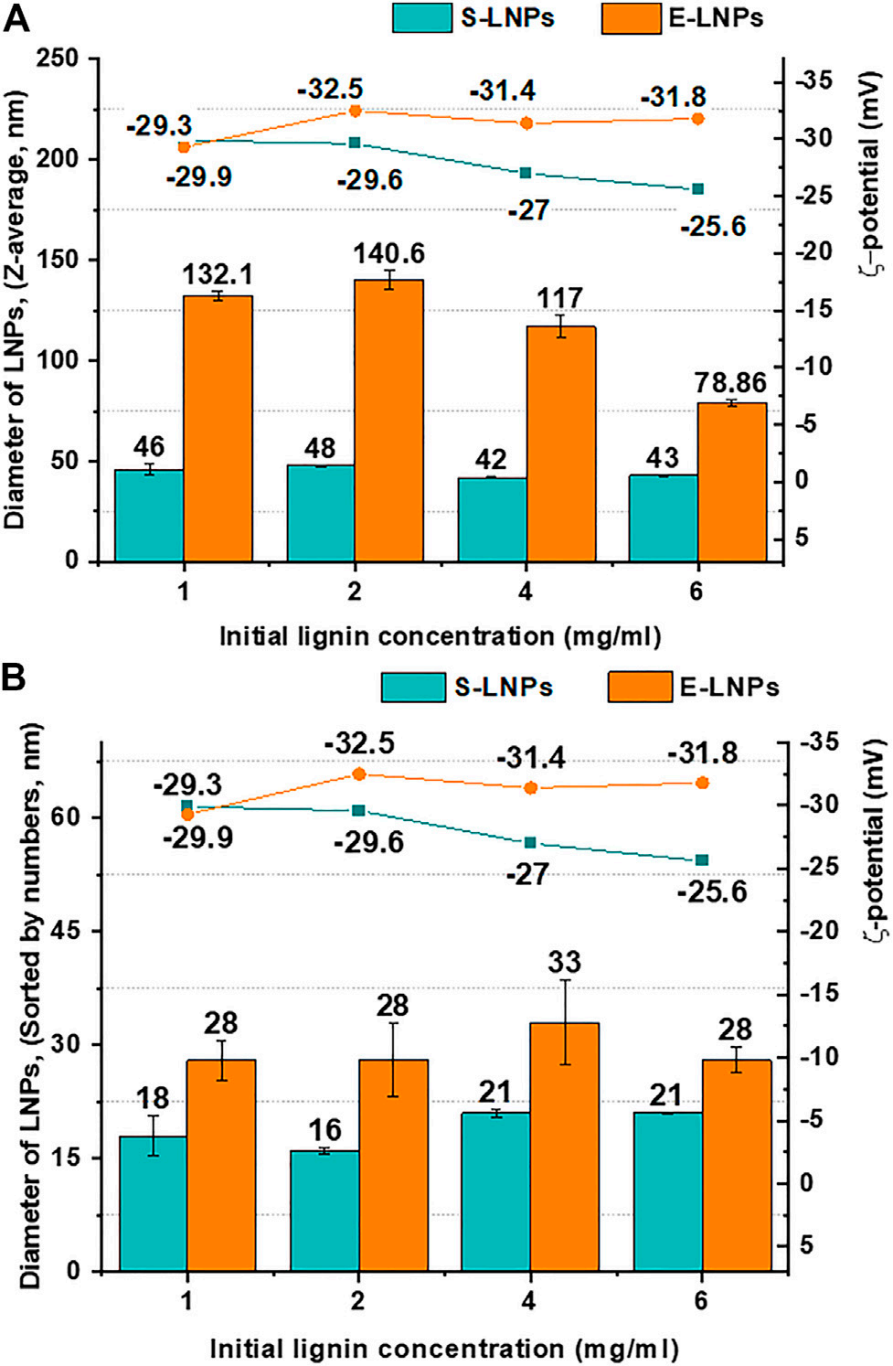
E-LNP size was larger than that of S-LNPs, and the size was not dependent on LNP concentration. Shown are **(A)** Z-average sizes (d, nm) and ζ-potential (mV) and **(B)** LNP sizes sorted by numbers (nm) and ζ-potential (mV) of the S- and E-LNPs obtained from the corresponding lignin fractions at the initial lignin concentrations between 1 and 6 mg/ml. Despite small variations of ζ-potential for S-LNPs, their surface charge was lower than that for E-LNPs.

**FIGURE 3:**
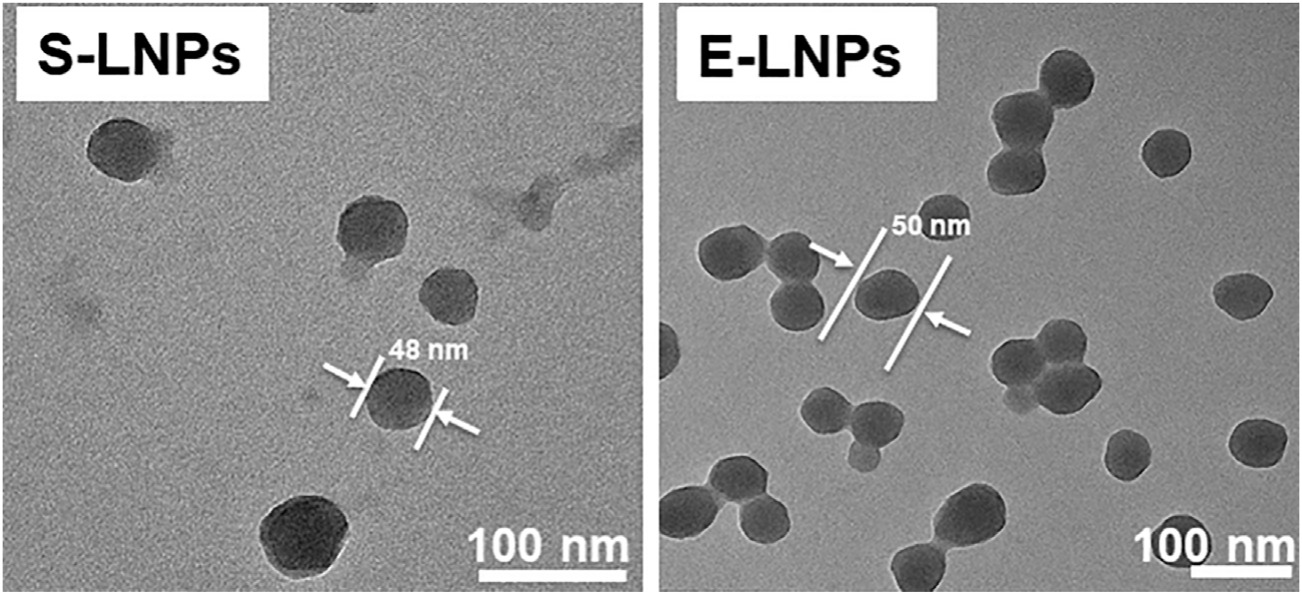
LNPs demonstrated a spherical form and a size of 48–60 nm as shown by TEM. The LNPs were synthesized at an initial lignin concentration of 1 mg/ml. Shown are TEM results for S- and E-LNPs.

Following fractionation, LNPs were obtained from spruce (S-LNPs) and eucalyptus (E-LNPs) using dialysis. Different pre-dialysis concentrations of lignins (1, 2, 4, and 6 mg/ml) were tested to identify their possible effect on the LNP size (Figure 2). In contrast to S-LNPs, the Zeta (Z)-average size of E-LNPs measured by dynamic light scattering (DLS) appeared to be larger, or close to 100 nm (Figure 2A). At the same time, using number distribution (sorting by numbers) gave rise to particle sizes of roughly 20 and 30 nm for S- and E-LNPs, respectively (Figure 2B). However, the TEM results showed that the real particle size of E-LNPs was ca 50 nm (Figure 3). The large LNP size determined by DLS was likely caused by a high sample PDI, which has been reported by several other laboratories to disrupt DLS measurements (Souza et al., 2016). TEM results correlated closest to the number distribution from light scattering.^1^

As it has been already shown in previous studies (Lievonen et al., 2016; Pylypchuk et al., 2021), the size of LNPs is usually proportional to the pre-dialysis lignin concentrations. However, using the acetone-insoluble fraction of lignin (fraction N5), the LNP size was shown to be independent of initial pre-dialysis lignin concentrations (Figure 2B). Following the number distribution of S- and E-LNP diameters shown in Figure 2B, we could see that despite the increase in concentration from 1 to 6 mg/ml, the LNP size remained relatively constant. This is most probably due to strong intramolecular π–π sandwich stacking interactions in the lignin macromolecule (Jawerth et al., 2020). In addition, acetone-insoluble spruce and eucalyptus lignin fractions demonstrated ∼4× times higher carbohydrate content (Figure 1B), than reported in other lignin fractions with lower M_*w*_ (Tagami et al., 2019), which could have an impact on the LNP self-assembly (designated as a carbohydrate-mediated LNP size growth inhibitor). The elevated carbohydrate content could hinder the ability of aromatic rings from other lignin molecules to interact with each other and thus prevent the growth of particles with increased lignin concentrations for both spruce and eucalyptus lignin. This carbohydrate-mediated size growth inhibitor can be considered as a hallmark feature of acetone-insoluble lignin fraction N5.

We further investigated the LNP electrophoretic mobility in aqueous solution, usually referred to as particle surface charge. Surface charge is one of the main parameters that influence the surface properties of NPs and can affect the phagocytosis of NPs in the blood circulation (Honary and Zahir, 2013). It was reported that NPs with higher surface charge bound more strongly to the cell membrane and show a higher cellular uptake (Honary and Zahir, 2013). Moreover, Levchenko et al. (2012) reported that the clearance rate of NPs from the blood is higher for negatively charged particles. In our study, we tested the ζ-potential, which is associated with the LNP surface charge and found that the ζ-potential of S-LNPs slightly decreased in absolute terms when the concentration of the initial lignin increased (Figure 2). In contrast, for E-LNPs the ζ-potential remained almost constant following an increase in lignin concentration (Figure 2, green and orange lines for S- and E-LNPs, respectively), making E-LNPs more stable, in accordance with the general interpretation of ζ-potential measurements (Shnoudeh et al., 2019).

We further performed SEM analysis of S- and E-LNPs and distinguished small particles with diameters between approx. 20 and 30 nm (Supplementary Figures S1, S2), which was in line with DLS measurements (Figures 2A,B). SEM further revealed that under the experimental conditions used, the LNPs from both lignin fractions tended to form sub-100-nm aggregates, which in turn assembled in larger conglomerates (Supplementary Figures S1, S2). According to the obtained TEM micrographs (Figure 3; Supplementary Figures S3, S4), the LNPs from both spruce and eucalyptus demonstrated a spherical shape with a diameter of about 50 nm. Notably, E-LNPs showed more interconnections between particles, whereas S-LNPs were more separated, as depicted in the SEM/TEM micrographs (Figure 3; Supplementary Figures S1–S4). This can potentially be due to a difference in surface properties of LNPs, as discussed in the NMR section below.

### NMR Analysis Showed the Presence of High Amounts of Carbohydrates on the Surface of Lignin Nanoparticles in Aqueous Suspensions

**FIGURE 4:**
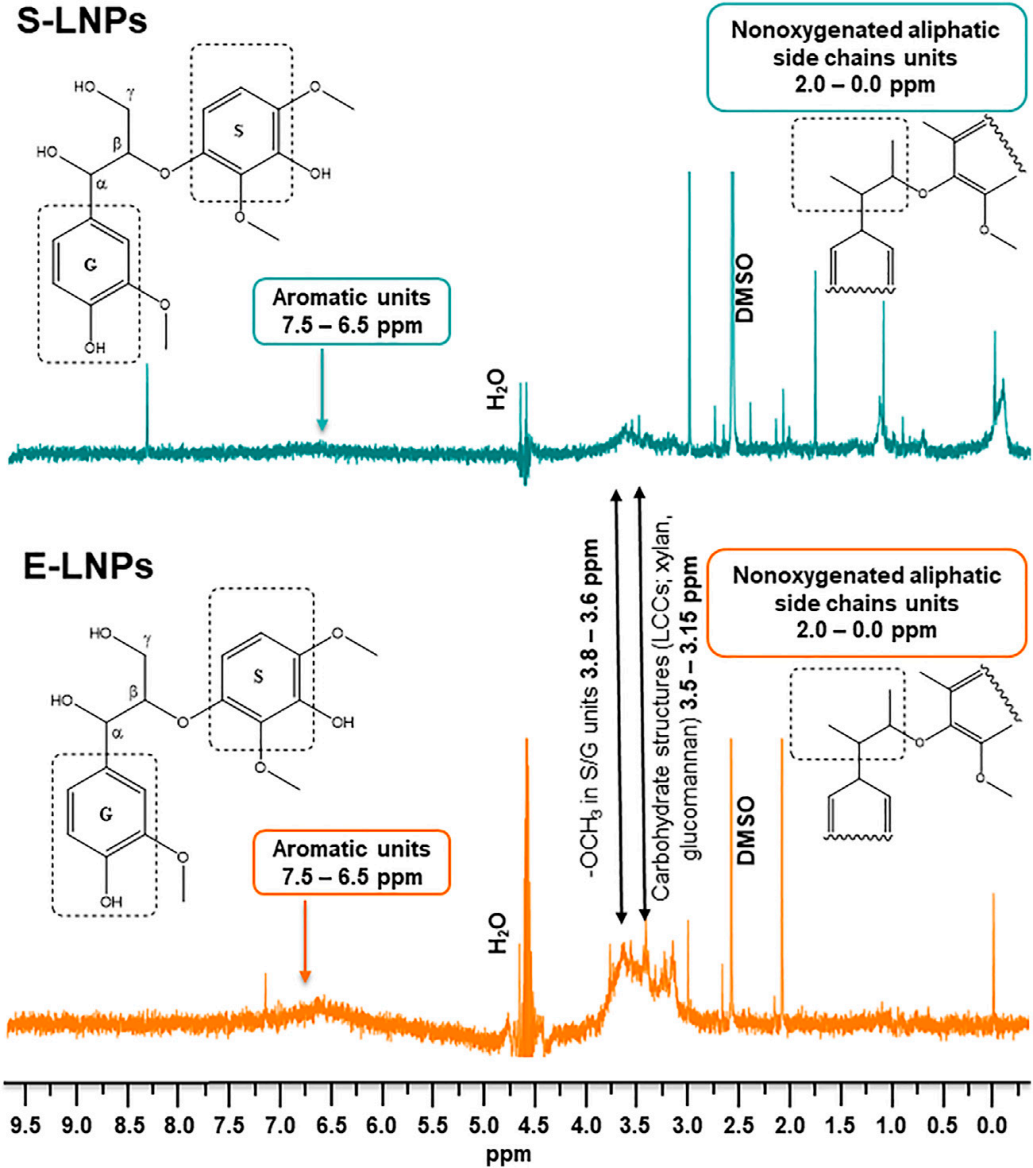
E-LNPs demonstrate the presence of higher numbers of carbohydrates on their surfaces as measured by liquid-state ^1^H NMR in aqueous suspensions. NMR revealed particular differences in chemical structures present on the surface of S- and E-LNPs: signals corresponding to carbohydrates and aromatic units were more pronounced in E-LNPs, whereas S-LNPs demonstrated more intense signal for nonpolar aliphatic side chains of lignin.

The investigation of LNP surface composition was performed by using the recently developed ^1^H NMR protocol (Pylypchuk et al., 2020). Briefly, the application of several NMR water suppression techniques in sequence on suspensions of LNPs made it possible to gain a clear insight into the surface composition of LNPs, since only the structures which interact with the solvent give a signal in the liquid-state NMR analysis (Figure 4). This NMR approach allowed for the direct investigation of the chemical groups present on the NP surface in aqueous suspensions (Zhou et al., 2008).

The aliphatic and carbohydrate contents for the original kraft lignin fractions as shown in Figures 1B,C correlated with the ^1^H NMR spectra in the aqueous suspension for the corresponding NPs, with a varying signal intensity among S- and E-LNPs (Figure 4).

When analyzing the ^1^H NMR spectra of LNPs, three regions of particular interest were selected: 1) the 3.15–3.5-ppm region, attributed to the presence of carbohydrates, such as xylan and glucomannan structures in lignin (Constant et al., 2016); 2) the 3.6–3.8-ppm region, attributed to methoxy groups of aromatic units; and 3) the 6.5–7.5-ppm region, attributed to aromatic protons.

**FIGURE 5:**
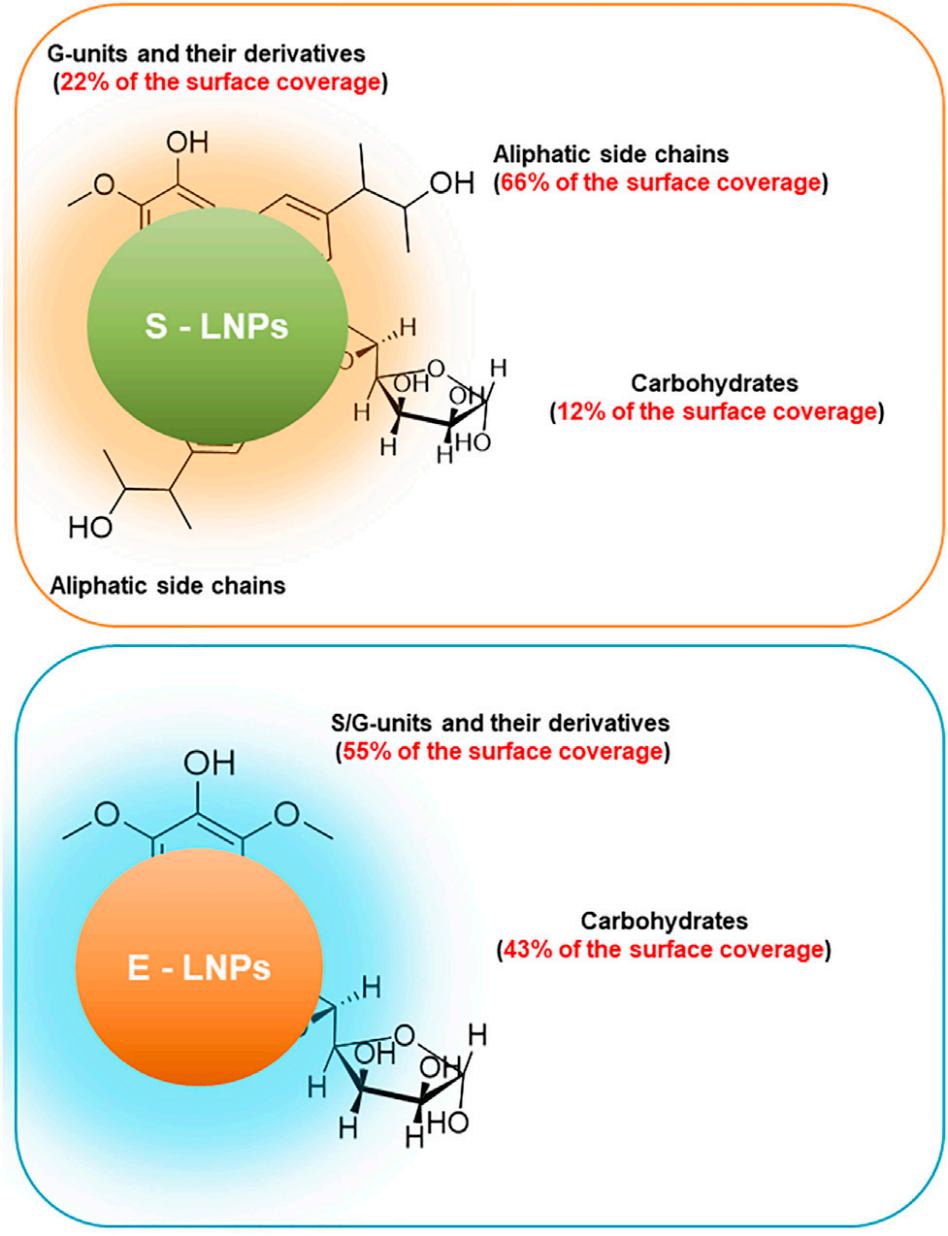
Surface of E-LNPs is covered mostly by S/G-units and carbohydrates, whereas surface of S-LNPs has three times lower carbohydrate content and G-units but is rich in aliphatic side chains. Schematic representation of the surface composition of LNPs from high-M_*w*_ lignin fractions. The ratio between aliphatic- and G-units was shown higher for S-LNPs, while the surface of E-LNPs contained an almost 1:1 ratio between carbohydrates and S/G aromatic units and was almost free of aliphatic side units of lignin.

The signal peak area in the 3.15–3.5-ppm range was observed to increase in E-LNPs, meaning that the surface of E-LNPs contains more carbohydrates than that of S-LNPs (Figure 4). This observation, however, is only valid for the lignin NPs. For the initial lignin fraction, the total carbohydrate content was instead determined to be higher for the spruce lignin-insoluble fraction (Figure 1B). Based on these results, we can conclude that the lower M_*w*_ of E-LNPs facilitated the lignin molecules’ self-assembly into LNPs and the re-distribution of lignin–carbohydrate complexes (LCCs) directly toward the E-LNP surface (Figure 5). Additionally, the signal intensity in the aromatic region (6.5–7.5 ppm) was higher in E-LNPs than in S-LNPs, allowing us to conclude that more aromatic protons were located on the E-LNP surface (Figure 4). For S-LNPs, however, more protons were detected in the non-polar aliphatic side-chain regions of lignin (0.0–2.0 ppm) as compared to E-LNPs (Figure 4). Finally, the intensity of signals originating from methoxy groups in S/G units (3.6–3.8 ppm) and aliphatic side chains (0.0–2.0 ppm) strongly indicated that the surface of E-LNPs was almost free from non-polar aliphatic residues, whereas on the surface of S-LNPs the hydrophobic non-polar aliphatic residues (e.g., hydroxypropenyl units) were dominating (Figure 5).

The ratio between aromatic/methoxy/non-oxygenated aliphatic protons is 0.48/1/4.46 for S-LNPs and 0.90/1/0.01 for E-LNPs (the full-range integrated ^1^H NMR spectra of the LNPs are presented in Supplementary Figures S5, S6). In this view, the surface of S-LNPs seems to possess more hydrophobic units than the surface of E-LNPs. A higher M_*w*_ of spruce lignin (≈20 kDa) and a higher content of aliphatic units, as compared to eucalyptus lignin, mediate the properties of S-LNPs, making their surfaces less polar. This higher content of aliphatic units also corresponds to the S-LNPs’ lower absolute values of surface charge in solution (ζ-potential, Figure 2). The visual representation of the most common chemical group on the LNP surface is presented in the scheme (Figure 5).

### Evaluation of the Lignin Nanoparticles Activity *In-Vitro*

After the characterization of LNPs, we tested the inhibitory capacity of LNP nanocompositions in cell culture experiments. S-LNPs and E-LNPs were incubated with two types of cancerous cells isolated either from primary HCC or from CCA tumors. Sorafenib for HCC and gemcitabine for CCA, as the standard therapeutics which are used for the treatment of these malignancies in the clinic, were added as a positive control. After the incubation with LNPs and therapeutics, several different tests, described below, were performed to define the inhibitory properties of LNPs in two types of liver cancer.

### Lignin Nanoparticles Demonstrated an Inhibitory Effect on the HCC Cell Line Shown in CVSA Experiments

**FIGURE 6:**
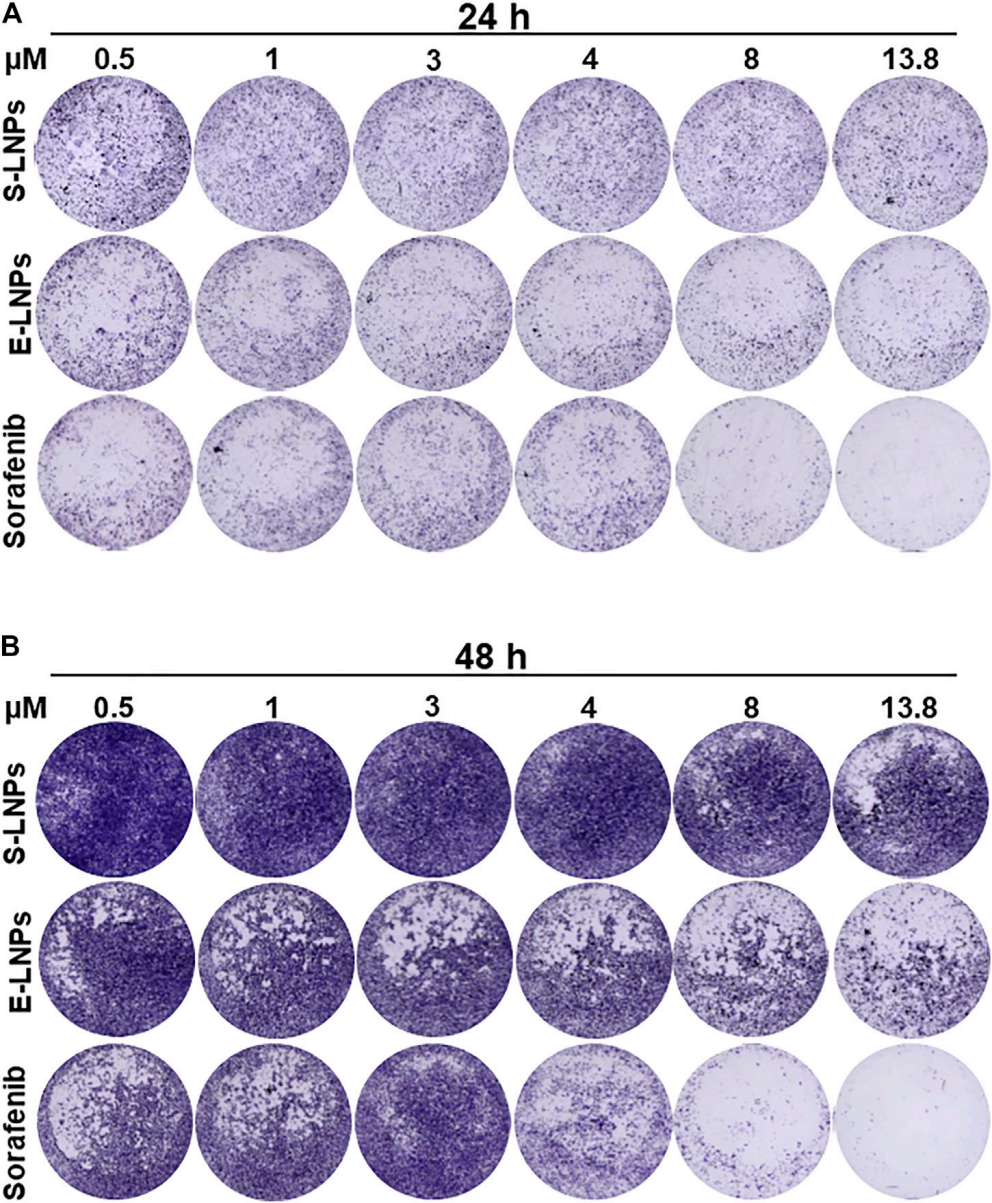
LNPs inhibited the growth of HCC in a dose-dependent manner. CVSA was performed in HCC cells after the treatment with S-LNPs and E-LNPs at different concentrations: 0.5, 1, 3, 4, 8, and 13.8 µM. Standard medicine sorafenib was used as a positive control. Further controls are shown in Supplementary Figure S7A. CVSA readouts were performed **(A)** 24 h and **(B)** 48 h post-incubation, respectively. For the direct comparison to a standard therapy, the sorafenib dose of 13.8 µm [plasma concentration, as reported for clinic (Fucile et al., 2015)] has been used as a positive control.

We first performed a CVSA that enables visualization of the inhibitory capacity of tested nanocomposites and therapeutics. S-LNPs did not significantly affect the growth of the HCC cell line (Figures 6A,B; Supplementary Figure S7A), and the growth inhibition was detected only at high doses of S-LNPs, in comparison to the sorafenib-treated group. In cells treated with E-LNPs, we detected a significant and dose-dependent decrease in cellular growth after 24 h of incubation (Figure 6A; Supplementary Figure S7A). The effect was even more pronounced after 48 h of incubation (Figure 6B; Supplementary Figure S7A). Interestingly, the inhibitory effect of S-LNPs and E-LNPs was observed exclusively in HCC and not in CCA cell lines (Supplementary Figures S7A,B). We further performed an analysis of CVSA microphotographs using ImageJ, which confirmed the inhibitory properties of E-LNPs in HCC, but not in CCA (Supplementary Figures S8, S9; Supplementary Table S2). Both controls, sorafenib and gemcitabine, showed a strong dose-dependent inhibitory effect on HCC and CCA cell lines, respectively, as expected (Figures 6A,B; Supplementary Figures S7–S9 and described in Fotopoulou et al. (2010), Kazim et al. (2015), Toyota et al. (2015), Kim et al. (2018), Wang et al. (2018), Zhang et al. (2018), and Iyer et al. (2019)). A lack of inhibition in the CCA cell line was probably associated with a more aggressive type of malignancy, due to the presence of two oncogenes and high metastatic potential of CCA. The latter still remains to be elucidated and/or verified in follow-up *in vivo* studies.

### Microscopy Showed the Presence of Yellowish Conglomerates in LNP-Treated Groups

**FIGURE 7:**
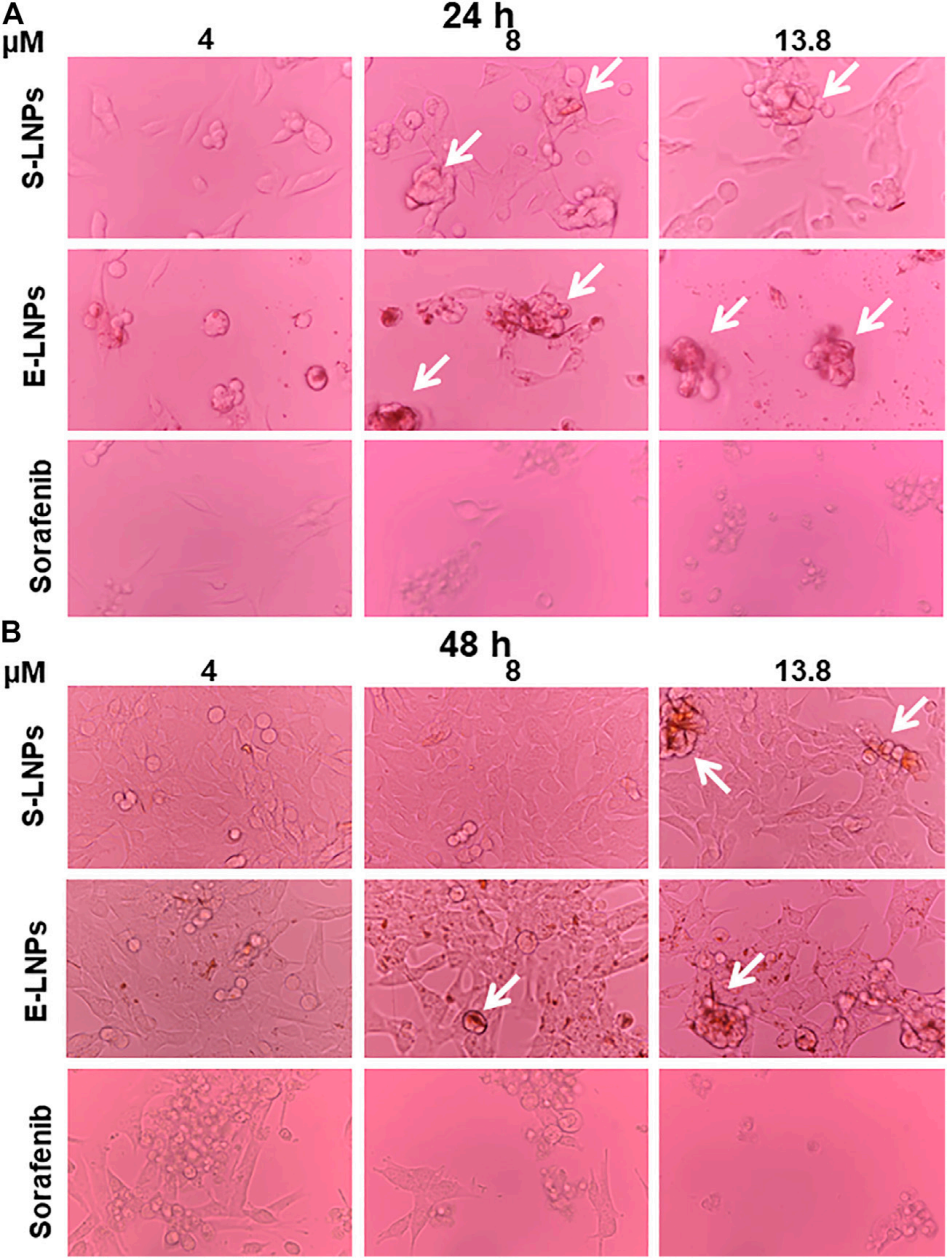
Microscopy showed a dose-dependent inhibition of HCC cells and accumulation of yellowish conglomerates in LNP-treated groups. HCC cells were treated with S-LNPs, E-LNPs, and standard therapy sorafenib. Bright-field microscopy (magnification ×40) was performed **(A)** 24 h and **(B)** 48 h post-incubation. Yellowish conglomerates are depicted with the white arrows. Details on all control groups are shown in Supplementary Figure S10.

The results obtained in CVSA correlated with the performed microscopy analysis. While performing a microscopical examination, we observed morphological changes in HCC cells after 24 h of incubation with S-LNP and E-LNP fractions (Figure 7A). Notably, in the E-LNP-treated group we detected yellowish conglomerates which occurred when high concentrations (ranging from 8 to 13.8 µM) of LNPs were added to the cells (conglomerates depicted with white arrows in Figures 7A,B). The sorafenib-treated group demonstrated no such conglomerates independent on the dose (Figures 7A,B; Supplementary Figure S10).

The decrease in cell counts in CCA microscopy correlated with CVSA results in gemcitabine-treated groups (Supplementary Figures S7, S11). The cell morphology and growth of the CCA cell line did not change significantly after treatment with LNPs, despite the formation of yellowish conglomerates (Supplementary Figure S11). None of the standard therapeutics induced the development of yellowish conglomerates (Figure 7; Supplementary Figures S10, S11). Therefore, the development of these conglomerates was considered a further hallmark of LNPs.

To check the hypothesis that the formation of the yellowish aggregates could be caused by the LNP aggregation due to the incubation at the elevated temperatures (37°C), a control experiment using DLS analysis was performed for 96 h in water (Supplementary Figure S12A). This analysis clearly demonstrated that conglomerates appeared mostly at the later time points (24–48 h, Figure 7; Supplementary Figures S10–S12). Aggregation was expected for both types of LNPs due to the increased Brownian motion at elevated temperatures. DLS analysis demonstrated heating at 37°C led to a significant increase in aggregate size, correlating with bright-field microscopy data (Figure 7; Supplementary Figures S10, S11, S12A). The capacity to form aggregates under increased temperatures could potentially be associated with the elevated carbohydrate content on the E-LNP surface. Moreover, the PDI of the suspensions slightly decreased while being incubated for longer time periods (Supplementary Figure S12B). From the TEM images, one can observe the unreacted lignin in form of aggregates (Figure 3; Supplementary Figures S3, S4, gray amorphous structures next to E-LNPs). These lignin aggregates cause a large PDI for E-LNP suspensions in the DLS experiment (Supplementary Figure S12B). In our opinion, the heating of LNP suspensions led to the decrease in the PDIs due to the consumption of unreacted lignin aggregates into the LNPs, leading to a slight increase in particle size.

Thus, considering the temperature tests, we propose that the presence of yellowish aggregates indicates that the LNPs are interacting with the cells. Since this effect manifested mostly in E-LNP-treated cell lines, which are more polar than S-LNPs, we suggest that E-LNPs are more likely to interact with cells in aqueous media resulting in LNPs transforming to yellowish aggregates.

### E-LNPs Demonstrated an Inhibitory Effect on the HCC Cell Line in CCK-8 Analysis

The results of CVSA and microscopy were further verified in CCK-8 assay and showed that E-LNPs inhibited the proliferation of HCC cells in comparison to the carrier (DMSO and water). The inhibitory effect in the E-LNP group was comparable to the sorafenib group at the 4-µM treatment dose (Supplementary Figure S13).

### E-LNPs Induced Late Apoptosis and Necroptosis While Inhibiting the HCC Cell Line as Detected *via* FACS Analysis

**FIGURE 8:**
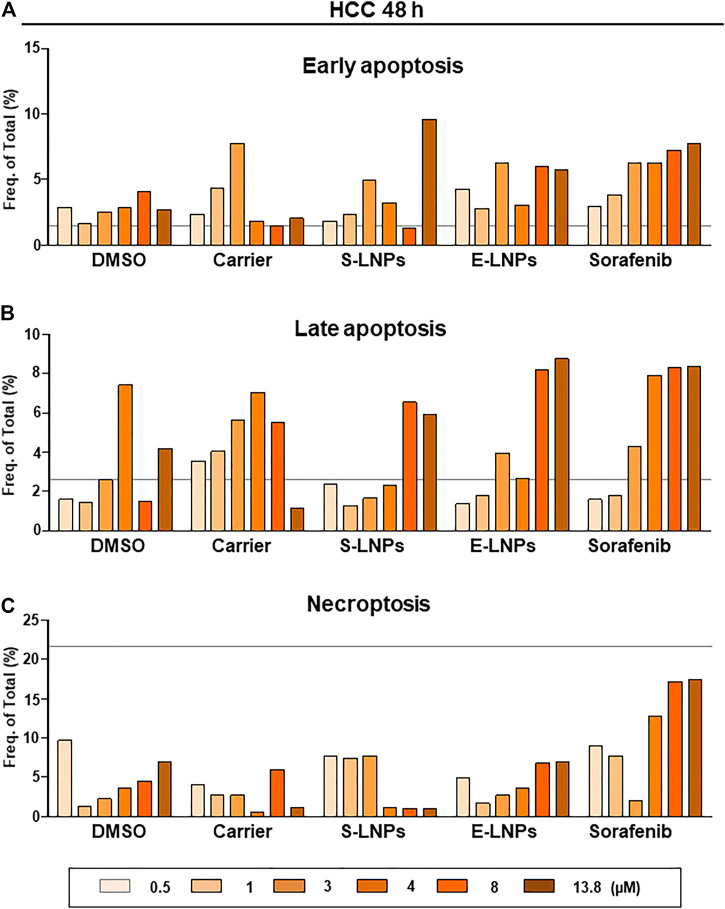
FACS analysis performed 48 h after the incubation with E-LNPs confirmed the induction of late apoptosis and necroptosis in the E-LNP-treated group. FACS analysis to detect **(A)** early and **(B)** late apoptosis and **(C)** necroptosis was performed in groups treated with S-LNP and E-LNP fractions and standard therapeutic sorafenib using the gating strategy depicted in Supplementary Figure S14. Shown are frequencies of **(A)** early and **(B)** late apoptotic and **(C)** necroptotic cells in percent. The gray line represents the values for the control group DMEM.

We further investigated the mechanism underlying the inhibition of the HCC cell line by the LNPs. To define the mechanism, we performed FACS analysis aiming to detect early and late apoptosis, as well as necroptosis (gating strategy is shown in Supplementary Figures S1, S2). After 48 h of incubation (Figures 8A–C), we observed a dose-dependent early apoptosis development in the sorafenib-treated group (Figure 8A). S- and E-LNP-treated cells showed varying numbers or early apoptosis, which were still higher than in the carrier-treated group (Figure 8A). However, late apoptosis was clearly shown upregulated in all three treatment groups. In particular, all high doses of S-LNPs and E-LNPs resulted in induction of late apoptosis (Figure 8B). Furthermore, the E-LNP-treated group showed the highest rate of late apoptosis, which was comparable to the sorafenib-treated group at concentrations 8 and 13.8 µM (Figure 8B). Interestingly, in E-LNP-treated cells we also detected elevated levels of necroptosis (a six times increase in comparison to the carrier at the 13.8-µM dose was observed), whereas the S-LNP-treated group did not show the presence of necroptosis at these treatment doses (Figure 8C). The sorafenib-treated group showed the highest necroptosis at 48 h of incubation (Figure 8C, 2.5 times increase detected at the 13.8-µM dose in comparison to DMSO as the carrier of sorafenib), and the result fully correlated with CVSA and microscopy, described above.

In the CCA cell line at 48 h post-incubation, gemcitabine clearly induced early as well as late apoptosis, whereas S-LNPs and E-LNPs induced mostly necroptosis (Supplementary Figures S15A–C demonstrates an 8 and 2 times increase for the gemcitabine 50-µM group in early and late apoptosis in comparison to the carrier (NaCl) and a 2.6 and 4.6 times increase for the S-LNP- and E-LNP-treated groups in necroptosis for the 13.8-µM dose in comparison to the respective carrier dose).

It is important to mention that our data on sorafenib standard therapy on HCC (induction of late apoptosis and necroptosis) is fully in line with previous reports (Lin et al., 2017). The same was also true for gemcitabine which induced early and late apoptosis in our and other studies (Pauwels et al., 2009; Li et al., 2020). Similar to sorafenib, both LNP types induced late apoptosis, whereas sorafenib and E-LNPs also additionally induced necroptosis in the HCC cell line. In CCA, LNPs in contrast to gemcitabine were able to induce mostly necroptosis. Gemcitabine induced early and late apoptosis and was herewith more efficient in inhibition of CCA growth than the LNPs. The results obtained in the first *in vitro* cytotoxicity experiments still have to be verified using follow-up IC_50_ and thorough toxicity studies *in vivo* in autochthonous/orthotopic HCC and CCA murine models.

### Lignin Nanoparticles in Comparison to Other Nanocomposites Used in Diagnostic and Treatment of Liver Cancer

The LNPs obtained in this study possess particular properties, which distinguish them from inorganic NPs that have been investigated previously for the treatment of liver cancer. In a previous research performed in HCC (Huh7 cell line), Kozenkova et al. investigated magnetite/Au hybrid nanostructures, such as Fe_3_O_4_-Au, and reported good magnetic, optical, and biocompatible properties and also possessed a low level of liver cancer cell cytotoxicity (Kozenkova et al., 2020). The average sizes of the Fe_3_O_4_-Au NPs were 12 ± 4 nm and 6 ± 1 nm for magnetite and Au counterparts, respectively (Kozenkova et al., 2020). Fe_3_O_4_-Au NPs were suggested as good candidates for theranostics of liver cancer and for the use as a magnetic resonance imaging (MRI)-contrast agent due to the high relaxometric efficiency and non-significant hepatotoxicity (Kozenkova et al., 2020). Due to the organic origin of LNPs, we, however, expect higher biocompatibility *in vivo*, than particles obtained using magnetite/Au hybrid nanostructures.

The shape, size, and surface charge of NPs are recognized as the most important parameters that play a role in particle interaction and surface-to-volume ratio changes, as recently reviewed for liver cancer (Taghizadeh et al., 2019). Au NPs have been shown to have homogeneous and heterogeneous catalytic activity and have attracted much attention, owing to their biocompatibility; specific bio-inertness, low cytotoxicity, ability to chemically modify their surface, and high surface-loading ability of drugs and genes and their superior imaging properties in positron emission tomography (PET), MRI, and computed tomography (CT) (Taghizadeh et al., 2019). Due to a high number of accessible functional groups on the surface of LNPs, as shown also in this study, LNPs can be easily modified to carry small and large molecules (proteins, chemotherapy, genotherapy, etc.). For instance, the hydration barrier and the ability for acid and base-catalyzed surface modifications of lignin oleate NPs have been recently described in a study by Moreno et al. (2021).

In a very interesting recent study, Liu et al. (2020) showed in a preclinical woodchuck model of hepatitis-induced HCC that the liver and spleen were the primary organs that sequester 60-nm Au NPs. In addition, Au NPs were shown to accumulate mostly in HCC periphery and less in the tumor core (Liu et al., 2020). The latter finding was concluded to be due to the lower abundance of macrophages penetrating into the tumor and the ability of macrophages to sequester injected NPs (Liu et al., 2020). Due to the high concentration of carbohydrates on the surface of LNPs in our study, we expect that they will efficiently attract macrophages toward the tumor core and activate T cells, which will be a key point in future HCC therapy design; however, the latter remains to be experimentally confirmed (Liu et al., 2020). In contrast to the study of Liu et al. (2020), our study shows that cancerous hepatocytes themselves appeared to take up 16–60-nm LNPs and we also observed an extracellular conglomerate formation.

In terms of CCA, a study by Kwak et al. reported a poly(D,L-lactide-co-glycolide) nanocomposite, consisting of a drug vorinostat and near-infrared dye-incorporated NPs (NIR-NPs). These nanocomposites exhibited spherical shapes with sizes <100 nm and represented a promising vehicle for targeted chemotherapy in HuCC-T1 CCA cells (Kwak et al., 2015). Interestingly, and in line with our data, empty NIR-NPs had no effect on tumor growth (Kwak et al., 2015). NIR-dye-incorporated NPs were intensively accumulated in the tumor region rather than in healthy tissue (Kwak et al., 2015). Vorinostat-NIR-NPs showed improved antitumor activity due to NIR-NPs which remained in the tumor tissue longer (low clearance capacity) than did free NIR dye (Kwak et al., 2015). Also, magnetic NPs were reported to be a useful device for the diagnosis and effective treatment of CCA, thereby using NPs as delivery platforms of chemotherapeutic agents (Tang et al., 2007; Lee et al., 2008; Helio et al., 2011).

### Potential Mechanism of the LNP Interaction With Liver Cancer Cells—Comparison of Our Results to the Recent Achievements in the Treatment of Liver Cancer With Other Polymeric NPs

Very few publications relate the biological behavior of lignin to certain biological processes (Gordobil et al., 2019). The biological activity and emerging role of lignin have been summarized in a review of Shu et al. (2021). Antitumor properties of eucalyptus-derived phytochemicals were related to the phenolic constituents and were summarized in a review (Abiri et al., 2021). Thá et al. (2021) demonstrated that eucalyptus kraft lignin, despite its strong antioxidant effect, can be responsible for the generation of significant levels of intracellular reactive oxygen species and cause oxidative damage to DNA of HepG2 cells.

The activity of lignin in cancer suppression can be linked to the presence of carbohydrates. In general, the hydrophilic nature of the polymer and the presence of sugar-like molecules in its structure promote the adhesion of the particles to the mucus (Mythri et al., 2011). The mucoadhesive properties of curcumin-cross-linked cellulose were investigated in the work of Hanafy et al. (2020), wherein it was found that strong mucoadhesive properties inhibit the growth of cancer cell lines at both programmed and non-programmed stages (Huh7 and HepG2 human liver cancer cell lines). Additionally, in a work by Wang et al. (2015) two high-M_*w*_, carbohydrate-rich (∼20% of carbohydrates) lignin polymers were investigated for their anti-proliferative properties. Similar to our study, the investigated lignin polymers induced cell death in a concentration-dependent manner, while apoptosis induction was largely cell-cycle independent. The authors also demonstrated that their lignin inhibited the activation of the nuclear transcription factor (NF-κB) in cancer cells. In a work of Rai et al. (2021), kraft lignin was combined with dextran polysaccharide in treatment of the colon cancer cells. It was concluded that the combination of the lignin with other biopolymers can become an alternative chemotherapeutic approach for malignant tumor treatments. Huang et al. (2018) reported that LCCs from bamboo inhibited the growth of MCF-7 breast cancer.

It must be said that our results are complimentary with this theory. The surface of the E-LNPs turned out to be more carbohydrate-rich and polar than that of S-LNPs, thus exhibiting better interaction with HCC cells. In addition to the influence of carbohydrates, we believe that the higher content of aliphatic side chains on the surface of S-LNPs hinders their interaction with cells. This finding agrees with studies by Moradipour (2021), who investigated the penetration of lignin compounds into model cell membranes. It was shown that hydroxypropenyl units in the lignin structure may present a barrier for penetration of the lignin molecules into the cell.

Considering the discussion above, we can relate the difference in the cytotoxic activity of the LNPs to the differences in the chemical structures presented on their surface. On the one hand, we can assign the enhanced activity of E-LNPs to the elevated content of the carbohydrates on their surface, favoring their interaction with cells. On the other hand, the elevated abundance of the hydroxypropenyl units on the surface of S-LNP hampers their ability to interact with cells. This conclusion is reflected in Graphical Abstract which summarizes the differences in the surface chemistry of the LNPs. These differences influence the LNPs’ interaction with cancerous cells and overall biotoxicity of the LNPs.

However, other mechanisms that can be both complementary and/or competitive can be considered for HCC. For instance, chitosan-grafted oleic acid was successfully used as a platform to deliver bromopyruvate, inhibiting glycolysis in HCC cells, thus causing their death (Hanafy et al., 2018). In another work, Hanafy et al. have shown that inhibition of HCC cells can be achieved through targeting DNA and RNA inside of the polymer-protein carrier (Hanafy et al., 2017). Glycol chitosan NPs incorporating all-trans retinoic acid were demonstrated to be effective in inhibiting the invasion, migration, and proliferation of human CCA cells, while empty glycol chitosan vehicles did not affect the viability of HuCC-T1 CCA cells (Chung et al., 2012), similar to our LNPs.

Towata et al. reported that hybrid liposomes were specifically accumulating in human CCA cells and induced G_1_ phase cell cycle arrest in CCA, which is known to be highly resistant to apoptosis (Towata et al., 2010). We, however, showed that LNPs could induce necroptosis in CCA, which was not sufficient to inhibit CCA growth.

## Conclusion

Lignin self-assembly and the formation of LNPs depend on the initial lignin chemical structure, namely, the M_*w*_, the number of methoxy groups, and the degree of cross-linking (Mishra and Ekielski, 2019). The lower M_*w*_ and different chemical structures (presence of S/G units with a higher number of methoxy groups) of lignin in eucalyptus wood as compared to lignin in spruce wood result in a less condensed structure of the lignin molecule. After the kraft pulping process, eucalyptus lignin molecules possess less carbon–carbon bonds between aromatic units and a higher number of aliphatic side chains (Ratnaweera et al., 2015; Tagami et al., 2019). The composition of carbohydrates is also different for hardwood (eucalyptus) and softwood (spruce), while the xylan content is much higher in eucalyptus. As a result, an elevated xylan content present in the high-M_*w*_ fraction of kraft lignin may have enhanced the cytotoxic properties of E-LNPs. Several factors, namely, the number of methoxy groups, carbohydrate content, cross-linking degree, shape, and surface structure, all impact the interactions of LNP with biological fluids and eukaryotic cells (**Graphical Abstract**). Our data on LNP surface properties, analyzed by NMR, DLS, and TEM, clearly demonstrated that hydrophilic E-LNPs more efficiently inhibited the growth of aggressive HCC cells, causing apoptosis of cancerous cells, than less polar S-LNPs. The differences in the surface chemistry of the LNPs influence their interaction with cancerous cells and mediate the increased biotoxicity of the E-LNPs. The enhanced activity of E-LNPs can be assigned to the elevated carbohydrate content on their surface, favoring their interaction with cells. The elevated abundance of the hydroxypropenyl units on the surface of S-LNP seems to hamper their ability to interact with cells. However, despite the higher polarity of E-LNPs, they did not induce apoptosis in CCA. Both LNP types were unable to inhibit CCA and induced only necroptosis. Despite the disability of LNPs to kill CCA cell lines, we do not exclude that a combination of LNPs and targeted therapeutics will be highly successful. Moreover, the complex structure of LNPs allows them to serve as delivery platforms or vehicles: LNPs can interact and incorporate molecules of interest and provide a controlled release of the molecules toward target cancerous cells (Sipponen et al., 2019). Due to a controlled dose-dependent cytotoxicity in HCC and no cytotoxicity in CCA, both types of LNPs represent ideal carrier platforms for therapeutics and combination thereof in aggressive liver malignancies. However, the latter remains to be thoroughly elucidated in the follow-up studies performing IC_50_ and toxicity studies *in vivo* and defining the most efficient therapeutic doses/regimes using HCC and CCA models.

## Data Availability

The original contributions presented in the study are included in the article and in Supplementary Material, further inquiries can be directed to the corresponding authors.

## Footnotes

1
https://www.malvernpanalytical.com/en

## References

[B1] Abiri R. Atabaki N. Sanusi R. Malik S. Abiri R. Safa P. (2021). New Insights into the Biological Properties of Eucalyptus-Derived Essential Oil: A Promising Green Anti-cancer Drug. Food Rev. Int., 1–36. 10.1080/87559129.2021.1877300

[B2] Argyropoulos D. S. (1994). Quantitative Phosphorus-31 NMR Analysis of Lignins, a New Tool for the Lignin Chemist. J. Wood Chem. Tech. 14, 45–63. 10.1080/02773819408003085

[B3] Banales J. M. Cardinale V. Carpino G. Marzioni M. Andersen J. B. Invernizzi P. (2016). Cholangiocarcinoma: Current Knowledge and Future Perspectives Consensus Statement from the European Network for the Study of Cholangiocarcinoma (ENS-CCA). Nat. Rev. Gastroenterol. Hepatol. 13, 261–280. 10.1038/nrgastro.2016.51 27095655

[B4] Banales J. M. Marin J. J. G. Lamarca A. Rodrigues P. M. Khan S. A. Roberts L. R. (2020). Cholangiocarcinoma 2020: the Next Horizon in Mechanisms and Management. Nat. Rev. Gastroenterol. Hepatol. 17, 557–588. 10.1038/s41575-020-0310-z 32606456 PMC7447603

[B5] Bhattacharya R. Mukherjee P. (2008). Biological Properties of "naked" Metal Nanoparticles☆. Adv. Drug Deliv. Rev. 60, 1289–1306. 10.1016/j.addr.2008.03.013 18501989

[B6] Brunt E. Aishima S. Clavien P.-A. Fowler K. Goodman Z. Gores G. (2018). cHCC-CCA: Consensus Terminology for Primary Liver Carcinomas with Both Hepatocytic and Cholangiocytic Differentation. Hepatology 68, 113–126. 10.1002/hep.29789 29360137 PMC6340292

[B7] Carlson C. M. Frandsen J. L. Kirchhof N. McIvor R. S. Largaespada D. A. (2005). Somatic Integration of an Oncogene-Harboring Sleeping Beauty Transposon Models Liver Tumor Development in the Mouse. Proc. Natl. Acad. Sci. 102, 17059–17064. 10.1073/pnas.0502974102 16286660 PMC1287966

[B8] Chung K.-D. Jeong Y.-I. Chung C.-W. Kim D. H. Kang D. H. (2012). Anti-tumor Activity of All-Trans Retinoic Acid-Incorporated Glycol Chitosan Nanoparticles against HuCC-T1 Human Cholangiocarcinoma Cells. Int. J. Pharmaceutics 422, 454–461. 10.1016/j.ijpharm.2011.10.057 22093956

[B9] Clements O. Eliahoo J. Kim J. U. Taylor-Robinson S. D. Khan S. A. (2020). Risk Factors for Intrahepatic and Extrahepatic Cholangiocarcinoma: A Systematic Review and Meta-Analysis. J. Hepatol. 72, 95–103. 10.1016/j.jhep.2019.09.007 31536748

[B10] Constant S. Wienk H. L. J. Frissen A. E. Peinder P. d. Boelens R. van Es D. S. (2016). New Insights into the Structure and Composition of Technical Lignins: a Comparative Characterisation Study. Green. Chem. 18, 2651–2665. 10.1039/c5gc03043a

[B11] Craig A. J. von Felden J. Garcia-Lezana T. Sarcognato S. Villanueva A. (2020). Tumour Evolution in Hepatocellular Carcinoma. Nat. Rev. Gastroenterol. Hepatol. 17, 139–152. 10.1038/s41575-019-0229-4 31792430

[B12] De Jong W. H. Borm P. J. (2008). Drug Delivery and Nanoparticles: Applications and Hazards. Ijn 3, 133–149. 10.2147/ijn.s596 18686775 PMC2527668

[B13] DeOliveira M. L. Cunningham S. C. Cameron J. L. Kamangar F. Winter J. M. Lillemoe K. D. (2007). Cholangiocarcinoma. Ann. Surg. 245, 755–762. 10.1097/01.sla.0000251366.62632.d3 17457168 PMC1877058

[B14] Duval A. Vilaplana F. Crestini C. Lawoko M. (2016). Solvent Screening for the Fractionation of Industrial Kraft Lignin. Holzforschung 70, 11–20. 10.1515/hf-2014-0346

[B15] Figueiredo P. Ferro C. Kemell M. Liu Z. Kiriazis A. Lintinen K. (2017a). Functionalization of Carboxylated Lignin Nanoparticles for Targeted and pH-Responsive Delivery of Anticancer Drugs. Nanomedicine 12, 2581–2596. 10.2217/nnm-2017-0219 28960138

[B16] Figueiredo P. Lahtinen M. H. Agustin M. B. Carvalho D. M. Hirvonen S. P. Penttilä P. A. (2021). Green Fabrication Approaches of Lignin Nanoparticles from Different Technical Lignins: A Comparison Study. ChemSusChem 14, 4718–4730. 10.1002/cssc.202101356 34398512 PMC8596756

[B17] Figueiredo P. Lepland A. Scodeller P. Fontana F. Torrieri G. Tiboni M. (2020). Peptide-guided Resiquimod-Loaded Lignin Nanoparticles Convert Tumor-Associated Macrophages from M2 to M1 Phenotype for Enhanced Chemotherapy. Acta Biomater 133, 231–243. 10.1016/j.actbio.2020.09.038 33011297

[B18] Figueiredo P. Lintinen K. Kiriazis A. Hynninen V. Liu Z. Bauleth-Ramos T. (2017b). *In Vitro* evaluation of Biodegradable Lignin-Based Nanoparticles for Drug Delivery and Enhanced Antiproliferation Effect in Cancer Cells. Biomaterials 121, 97–108. 10.1016/j.biomaterials.2016.12.034 28081462

[B19] Figueiredo P. Sipponen M. H. Lintinen K. Correia A. Kiriazis A. Yli‐Kauhaluoma J. (2019). Preparation and Characterization of Dentin Phosphophoryn‐Derived Peptide‐Functionalized Lignin Nanoparticles for Enhanced Cellular Uptake. Small 15, 1901427. 10.1002/smll.201901427 PMC8042775 31062448

[B20] Fotopoulou C. Baumunk D. Schmidt S. C. Schumacher G. (2010). Additive Growth Inhibition after Combined Treatment of 2-Methoxyestradiol and Conventional Chemotherapeutic Agents in Human Pancreatic Cancer Cells. Anticancer Res. 30, 4619–4624. 21115915

[B21] Fucile C. Marenco S. Bazzica M. Zuccoli M. L. Lantieri F. Robbiano L. (2015). Measurement of Sorafenib Plasma Concentration by High-Performance Liquid Chromatography in Patients with Advanced Hepatocellular Carcinoma: Is it Useful the Application in Clinical Practice? A Pilot Study. Med. Oncol. 32, 335. 10.1007/s12032-014-0335-7 25429830

[B22] Gao W. Fatehi P. (2019). Lignin for Polymer and Nanoparticle Production: Current Status and Challenges. Can. J. Chem. Eng. 97, 2827–2842. 10.1002/cjce.23620

[B23] Gioia C. Colonna M. Tagami A. Medina L. Sevastyanova O. Berglund L. A. (2020). Lignin-Based Epoxy Resins: Unravelling the Relationship between Structure and Material Properties. Biomacromolecules 21, 1920–1928. 10.1021/acs.biomac.0c00057 32160463 PMC7997103

[B24] Gioia C. Lo Re G. Lawoko M. Berglund L. (2018). Tunable Thermosetting Epoxies Based on Fractionated and Well-Characterized Lignins. J. Am. Chem. Soc. 140, 4054–4061. 10.1021/jacs.7b13620 29498848

[B25] Giummarella N. Lindén P. A. Areskogh D. Lawoko M. (2020). Fractional Profiling of Kraft Lignin Structure: Unravelling Insights on Lignin Reaction Mechanisms. ACS Sustain. Chem. Eng. 8, 1112–1120. 10.1021/acssuschemeng.9b06027

[B26] Gordobil O. Oberemko A. Saulis G. Baublys V. Labidi J. (2019). *In Vitro* cytotoxicity Studies of Industrial Eucalyptus Kraft Lignins on Mouse Hepatoma, Melanoma and Chinese Hamster Ovary Cells. Int. J. Biol. macromolecules 135, 353–361. 10.1016/j.ijbiomac.2019.05.111 31125648

[B27] Granata A. Argyropoulos D. S. (1995). 2-Chloro-4,4,5,5-Tetramethyl-1,3,2-Dioxaphospholane, a Reagent for the Accurate Determination of the Uncondensed and Condensed Phenolic Moieties in Lignins. J. Agric. Food Chem. 43, 1538–1544. 10.1021/jf00054a023

[B28] Guerra A. Filpponen I. Lucia L. A. Argyropoulos D. S. (2006). Comparative Evaluation of Three Lignin Isolation Protocols for Various wood Species. J. Agric. Food Chem. 54, 9696–9705. 10.1021/jf062433c 17177489

[B29] Guo X. Shen W. (2020). Latest Evidence on Immunotherapy for Cholangiocarcinoma. Oncol. Lett. 20, 381. 10.3892/ol.2020.12244 33154779 PMC7608025

[B30] Gürlevik E. Fleischmann-Mundt B. Armbrecht N. Longerich T. Woller N. Kloos A. (2013). Adjuvant Gemcitabine Therapy Improves Survival in a Locally Induced, R0-Resectable Model of Metastatic Intrahepatic Cholangiocarcinoma. Hepatology 58, 1031–1041. 10.1002/hep.26468 23686746

[B31] Gürlevik E. Fleischmann-Mundt B. Brooks J. Demir I. E. Steiger K. Ribback S. (2016). Administration of Gemcitabine after Pancreatic Tumor Resection in Mice Induces an Antitumor Immune Response Mediated by Natural Killer Cells. Gastroenterology 151, 338–350. e337. 10.1053/j.gastro.2016.05.004 27210037

[B32] Hanafy N. A. Ferraro M. M. Gaballo A. Dini L. Tasco V. Nobile C. (2016). Fabrication and Characterization of ALK1fc-Loaded Fluoro-Magnetic Nanoparticles for Inhibiting TGF β1 in Hepatocellular Carcinoma. RSC Adv. 6, 48834–48842. 10.1039/c6ra06345d

[B33] Hanafy N. A. N. (20172017). “Development and Production of Multifunctional Bio Nanoengineered Drug Delivery Systems Loaded by TGFβ1 Inhibitors for Delivering into Hepatocellular Carcinoma Cells,”. Diss Ph D Thesis (Lecce, Italy: Salento University).

[B34] Hanafy N. A. N. Leporatti S. El-Kemary M. (2020). Mucoadhesive Curcumin Crosslinked Carboxy Methyl Cellulose Might Increase Inhibitory Efficiency for Liver Cancer Treatment. Mater. Sci. Eng. C 116, 111119. 10.1016/j.msec.2020.111119 32806233

[B35] Hanafy N. A. N. Quarta A. Di Corato R. Dini L. Nobile C. Tasco V. (2017). Hybrid Polymeric-Protein Nano-Carriers (HPPNC) for Targeted Delivery of TGFβ Inhibitors to Hepatocellular Carcinoma Cells. J. Mater. Sci. Mater. Med. 28, 120. 10.1007/s10856-017-5930-7 28685231

[B36] Hanafy N. Dini L. Citti C. Cannazza G. Leporatti S. (2018). Inihibition of Glycolysis by Using a Micro/Nano-Lipid Bromopyruvic Chitosan Carrier as a Promising Tool to Improve Treatment of Hepatocellular Carcinoma. Nanomaterials 8, 34. 10.3390/nano8010034 PMC5791121 29320411

[B37] Helio J. V. Braga K. I. Bluemke D. (2011). MR Imaging of Intrahepatic Cholangiocarcinoma Use of Ferumoxides for Lesion Localization and Extension. Am. J. Roentgenology 2011, 111–114. 10.2214/ajr.177.1.1770111 11418408

[B38] Hochnadel I. Kossatz-Boehlert U. Jedicke N. Lenzen H. Manns M. P. Yevsa T. (2017). Cancer Vaccines and Immunotherapeutic Approaches in Hepatobiliary and Pancreatic Cancers. Hum. Vaccin. Immunother. 13, 2931–2952. 10.1080/21645515.2017.1359362 29112462 PMC5718787

[B39] Honary S. Zahir F. (2013). Effect of Zeta Potential on the Properties of Nano-Drug Delivery Systems - A Review (Part 1). Trop. J. Pharm. Res. 12, 255–264. 10.4314/tjpr.v12i2.19

[B40] Huang C. Tang S. Zhang W. Tao Y. Lai C. Li X. (2018). Unveiling the Structural Properties of Lignin-Carbohydrate Complexes in Bamboo Residues and its Functionality as Antioxidants and Immunostimulants. ACS Sustain. Chem. Eng. 6, 12522–12531. 10.1021/acssuschemeng.8b03262

[B41] Hwang J. H. Choi C. W. Kim H. W. Kim D. H. Kwak T. W. Lee H. M. (2013). Dextran-b-poly(L-histidine) Copolymer Nanoparticles for Ph-Responsive Drug Delivery to Tumor Cells. Int. J. Nanomedicine 8, 3197–3207. 10.2147/IJN.S49459 23986636 PMC3754766

[B42] Iyer R. V. Maguire O. Kim M. Curtin L. I. Sexton S. Fisher D. T. (2019). Dose-Dependent Sorafenib-Induced Immunosuppression Is Associated with Aberrant NFAT Activation and Expression of PD-1 in T Cells. Cancers 11, 681. 10.3390/cancers11050681 PMC6562672 31100868

[B43] Jawerth M. E. Brett C. J. Terrier C. Larsson P. T. Lawoko M. Roth S. V. (2020). Mechanical and Morphological Properties of Lignin-Based Thermosets. ACS Appl. Polym. Mater. 2, 668–676. 10.1021/acsapm.9b01007

[B44] Johnston M. P. Khakoo S. I. (2019). Immunotherapy for Hepatocellular Carcinoma: Current and Future. Wjg 25, 2977–2989. 10.3748/wjg.v25.i24.2977 31293335 PMC6603808

[B45] Kang T.-W. Yevsa T. Woller N. Hoenicke L. Wuestefeld T. Dauch D. (2011). Senescence Surveillance of Pre-malignant Hepatocytes Limits Liver Cancer Development. Nature 479, 547–551. 10.1038/nature10599 22080947

[B46] Kaur R. Uppal S. K. Sharma P. (2017). Antioxidant and Antibacterial Activities of Sugarcane Bagasse Lignin and Chemically Modified Lignins. Sugar Tech. 19, 675–680. 10.1007/s12355-017-0513-y

[B47] Kazim S. Malafa M. P. Coppola D. Husain K. Zibadi S. Kashyap T. (2015). Selective Nuclear Export Inhibitor KPT-330 Enhances the Antitumor Activity of Gemcitabine in Human Pancreatic Cancer. Mol. Cancer Ther. 14, 1570–1581. 10.1158/1535-7163.mct-15-0104 25934708 PMC4577050

[B48] Kim Y.-S. Lee Y.-M. Oh T.-I. Shin D. Kim G.-H. Kan S.-Y. (2018). Emodin Sensitizes Hepatocellular Carcinoma Cells to the Anti-cancer Effect of Sorafenib through Suppression of Cholesterol Metabolism. Ijms 19, 3127. 10.3390/ijms19103127 PMC6213641 30321984

[B49] Korhonen J. Honkasalo A. Seppälä J. (2018). Circular Economy: the Concept and its Limitations. Ecol. Econ. 143, 37–46. 10.1016/j.ecolecon.2017.06.041

[B50] Kozenkova E. Levada K. Efremova M. V. Omelyanchik A. Nalench Y. A. Garanina A. S. (2020). Multifunctional Fe3O4-Au Nanoparticles for the MRI Diagnosis and Potential Treatment of Liver Cancer. Nanomaterials 10, 1646. 10.3390/nano10091646 PMC7558883 32825748

[B51] Kwak T. W. Kim D. H. Jeong Y.-I. Kang D. H. (2015). Antitumor Activity of Vorinostat-Incorporated Nanoparticles against Human Cholangiocarcinoma Cells. J. Nanobiotechnol 13, 60. 10.1186/s12951-015-0122-4 PMC4583727 26410576

[B52] Lee Y. Lee J. S. Kim C.-M. Jeong J. Y. Choi J.-I. Kim M. J. (2008). Area of Paradoxical Signal Drop after the Administration of Superparamagnetic Iron Oxide on the T2-Weighted Image of a Patient with Lymphangitic Metastasis of the Liver. Magn. Reson. Imaging 26, 577–582. 10.1016/j.mri.2007.10.012 18093780

[B53] Levchenko T. S. Hartner W. C. Torchilin V. P. (2012). Liposomes in Diagnosis and Treatment of Cardiovascular Disorders. Methodist Debakey Cardiovasc. J. 8, 36–41. 10.14797/mdcj-8-1-36 22891109 PMC3405779

[B54] Li W. Zhu Y. Zhang K. Yu X. Lin H. Wu W. (2020). PROM2 Promotes Gemcitabine Chemoresistance via Activating the Akt Signaling Pathway in Pancreatic Cancer. Exp. Mol. Med. 52, 409–422. 10.1038/s12276-020-0390-4 32123287 PMC7156657

[B55] Lievonen M. Valle-Delgado J. J. Mattinen M.-L. Hult E.-L. Lintinen K. Kostiainen M. A. (2016). A Simple Process for Lignin Nanoparticle Preparation. Green. Chem. 18, 1416–1422. 10.1039/c5gc01436k

[B56] Lin S. Hoffmann K. Gao C. Petrulionis M. Herr I. Schemmer P. (2017). Melatonin Promotes Sorafenib-Induced Apoptosis through Synergistic Activation of JNK/c-jun Pathway in Human Hepatocellular Carcinoma. J. Pineal Res. 62, e12398. 10.1111/jpi.12398 28178378

[B57] Liu L. Y. Ma X.-Z. Ouyang B. Ings D. P. Marwah S. Liu J. (2020). Nanoparticle Uptake in a Spontaneous and Immunocompetent Woodchuck Liver Cancer Model. ACS Nano 14, 4698–4715. 10.1021/acsnano.0c00468 32255624

[B58] Llovet J. M. Ricci S. Mazzaferro V. Hilgard P. Gane E. Blanc J.-F. (2008). Sorafenib in Advanced Hepatocellular Carcinoma. N. Engl. J. Med. 359, 378–390. 10.1056/nejmoa0708857 18650514

[B59] Mishra P. K. Ekielski A. (2019). The Self-Assembly of Lignin and its Application in Nanoparticle Synthesis: A Short Review. Nanomaterials (Basel) 9, 1–15. 10.3390/nano9020243 PMC6410071 30754724

[B60] Moghadam F. F. (2017). Using Nanoparticles in Medicine for Liver Cancer Imaging. Oman Med. J. 32, 269–274. 10.5001/omj.2017.54 28804578 PMC5534229

[B61] Moradipour M. (2021). Interactions of Lignin Dimers with Engineered Surfaces and Model Cell Membranes for Design of Lignin-Based Materials. Theses Dissertations-Chemical Mater. Eng. 135.

[B62] Moreno A. Liu J. Gueret R. Hadi S. E. Bergström L. Slabon A. (2021). Unravelling the Hydration Barrier of Lignin Oleate Nanoparticles for Acid- and Base-Catalyzed Functionalization in Dispersion State. Angew. Chem. Int. Edition 60, 20897–20905. 10.1002/anie.202106743 PMC8518943 34196470

[B63] Mythri G. Kavitha K. Rupesh M. Singh J. S. D. (2011). Novel Mucoadhesive Polymers –A Review. JAPS 01, 37–42.

[B64] Nakeeb A. Pitt H. A. Sohn T. A. Coleman J. Abrams R. A. Piantadosi S. (1996). Cholangiocarcinoma. Ann. Surg. 224, 463–475. discussion 473-465. 10.1097/00000658-199610000-00005 8857851 PMC1235406

[B65] Nenu I. Breaban I. Pascalau S. Bora C.-N. Stefanescu H. (2018). The Future Is Now: beyond First Line Systemic Therapy in Hepatocellular Carcinoma. Translational Cancer Res. 8, S261–S274. 10.21037/tcr.2018.11.23 PMC8797356 35117106

[B66] Olsén P. Jawerth M. Lawoko M. Johansson M. Berglund L. A. (2019). Transforming Technical Lignins to Structurally Defined star-copolymers under Ambient Conditions. Green. Chem. 21, 2478–2486. 10.1039/c9gc00835g

[B67] Österberg M. Sipponen M. H. Mattos B. D. Rojas O. J. (2020). Spherical Lignin Particles: a Review on Their Sustainability and Applications. Green. Chem. 22, 2712–2733. 10.1039/d0gc00096e

[B68] Pan X. Kadla J. F. Ehara K. Gilkes N. Saddler J. N. (2006). Organosolv Ethanol Lignin from Hybrid poplar as a Radical Scavenger: Relationship between Lignin Structure, Extraction Conditions, and Antioxidant Activity. J. Agric. Food Chem. 54, 5806–5813. 10.1021/jf0605392 16881681

[B69] Pauwels B. Vermorken J. B. Wouters A. Ides J. Van Laere S. Lambrechts H. A. J. (2009). The Role of Apoptotic Cell Death in the Radiosensitising Effect of Gemcitabine. Br. J. Cancer 101, 628–636. 10.1038/sj.bjc.6605145 19672265 PMC2736812

[B70] Ponomarenko J. Dizhbite T. Lauberts M. Volperts A. Dobele G. Telysheva G. (2015). Analytical Pyrolysis - A Tool for Revealing of Lignin Structure-Antioxidant Activity Relationship. J. Anal. Appl. Pyrolysis 113, 360–369. 10.1016/j.jaap.2015.02.027

[B71] Pylypchuk I. V. Lindén P. A. Lindström M. E. Sevastyanova O. (2020). New Insight into the Surface Structure of Lignin Nanoparticles Revealed by 1H Liquid-State NMR Spectroscopy. ACS Sustain. Chem. Eng. 8, 13805–13812. 10.1021/acssuschemeng.0c05119

[B72] Pylypchuk I. V. Riazanova A. Lindström M. E. Sevastyanova O. (2021). Structural and Molecular-Weight-Dependency in the Formation of Lignin Nanoparticles from Fractionated Soft- and Hardwood Lignins. Green. Chem. 23, 3061–3072. 10.1039/d0gc04058d

[B73] Rai S. Arun S. Kureel A. K. Dutta P. K. Mehrotra G. K. (2021). Preparation of Dextran Aldehyde and BSA Conjugates from Ligno-Cellulosic Biowaste for Antioxidant and Anti-cancer Efficacy. Waste Biomass Valor. 12, 1327–1339. 10.1007/s12649-020-01088-0

[B74] Ratnaweera D. R. Saha D. Pingali S. V. Labbé N. Naskar A. K. Dadmun M. (2015). The Impact of Lignin Source on its Self-Assembly in Solution. RSC Adv. 5, 67258–67266. 10.1039/c5ra13485d

[B75] Rudalska R. Dauch D. Longerich T. McJunkin K. Wuestefeld T. Kang T.-W. (2014). *In Vivo* RNAi Screening Identifies a Mechanism of Sorafenib Resistance in Liver Cancer. Nat. Med. 20, 1138–1146. 10.1038/nm.3679 25216638 PMC4587571

[B76] Shnoudeh A. J. Hamad I. Abdo R. W. Qadumii L. Jaber A. Y. Surchi H. S. (2019). “Synthesis, Characterization, and Applications of Metal Nanoparticles,” in Biomaterials and Bionanotechnology. Editor Tekade R. K. (Cambridge, Massachusetts: Academic Press), 527–612. 10.1016/b978-0-12-814427-5.00015-9

[B77] Shu F. Jiang B. Yuan Y. Li M. Wu W. Jin Y. (2021). Biological Activities and Emerging Roles of Lignin and Lignin-Based Products─A Review. Biomacromolecules 22, 4905–4918. 10.1021/acs.biomac.1c00805 34806363

[B78] Siddiqui L. Bag J. Seetha D. Mittal D. Leekha A. Mishra H. (2020). Assessing the Potential of Lignin Nanoparticles as Drug Carrier: Synthesis, Cytotoxicity and Genotoxicity Studies. Int. J. Biol. macromolecules 152, 786–802. 10.1016/j.ijbiomac.2020.02.311 32114178

[B79] Siddiqui L. Mishra H. Mishra P. K. Iqbal Z. Talegaonkar S. (2018). Novel 4-in-1 Strategy to Combat colon Cancer, Drug Resistance and Cancer Relapse Utilizing Functionalized Bioinspiring Lignin Nanoparticle. Med. hypotheses 121, 10–14. 10.1016/j.mehy.2018.09.003 30396459

[B80] Sipponen M. H. Lange H. Ago M. Crestini C. (2018). Understanding Lignin Aggregation Processes. A Case Study: Budesonide Entrapment and Stimuli Controlled Release from Lignin Nanoparticles. ACS Sustain. Chem. Eng. 6, 9342–9351. 10.1021/acssuschemeng.8b01652 30271691 PMC6156105

[B81] Sipponen M. H. Lange H. Crestini C. Henn A. Österberg M. (2019). Lignin for Nano‐ and Microscaled Carrier Systems: Applications, Trends, and Challenges. ChemSusChem 12, 2039–2054. 10.1002/cssc.201900480 30933420 PMC6593669

[B82] Śliwka L. Wiktorska K. Suchocki P. Milczarek M. Mielczarek S. Lubelska K. (2016). The Comparison of MTT and CVS Assays for the Assessment of Anticancer Agent Interactions. PLoS One 11, e0155772. 10.1371/journal.pone.0155772 27196402 PMC4873276

[B83] Souza T. G. F. Ciminelli V. S. T. Mohallem N. D. S. (2016). A Comparison of TEM and DLS Methods to Characterize Size Distribution of Ceramic Nanoparticles. J. Phys. Conf. Ser. 733, 012039. 10.1088/1742-6596/733/1/012039

[B84] Tagami A. Gioia C. Lauberts M. Budnyak T. Moriana R. Lindström M. E. (2019). Solvent Fractionation of Softwood and Hardwood Kraft Lignins for More Efficient Uses: Compositional, Structural, thermal, Antioxidant and Adsorption Properties. Ind. Crops Prod. 129, 123–134. 10.1016/j.indcrop.2018.11.067

[B85] Taghizadeh S. Alimardani V. Roudbali P. L. Ghasemi Y. Kaviani E. (2019). Gold Nanoparticles Application in Liver Cancer. Photodiagnosis Photodynamic Ther. 25, 389–400. 10.1016/j.pdpdt.2019.01.027 30684673

[B86] Tang T. Zheng J. W. Chen B. Li H. Li X. Xue K. Y. (2007). Effects of Targeting Magnetic Drug Nanoparticles on Human Cholangiocarcinoma Xenografts in Nude Mice. Hepatobiliary Pancreat. Dis. Int. 6, 303–307. 17548256

[B87] Thá E. L. Matos M. Avelino F. Lomonaco D. Rodrigues-Souza I. Gagosian V. S. C. (2021). Safety Aspects of Kraft Lignin Fractions: Discussions on the in Chemico Antioxidant Activity and the Induction of Oxidative Stress on a Cell-Based *In Vitro* Model. Int. J. Biol. Macromolecules 182, 977–986. 10.1016/j.ijbiomac.2021.04.103 33887289

[B88] Tomani P. (2010). The Lignoboost Process. Cellulose Chem. Technol. 44, 53–58.

[B89] Towata T. Komizu Y. Kariya R. Suzu S. Matsumoto Y. Kobayashi N. (2010). Hybrid Liposomes Inhibit the Growth of Cholangiocarcinoma by Induction of Cell Cycle Arrest in G1 Phase. Bioorg. Med. Chem. Lett. 20, 3680–3682. 10.1016/j.bmcl.2010.04.091 20494578

[B90] Toyota Y. Iwama H. Kato K. Tani J. Katsura A. Miyata M. (2015). Mechanism of Gemcitabine-Induced Suppression of Human Cholangiocellular Carcinoma Cell Growth. Int. J. Oncol. 47, 1293–1302. 10.3892/ijo.2015.3118 26252371

[B91] Walcher L. Kistenmacher A.-K. Suo H. Kitte R. Dluczek S. Strauß A. (2020). Cancer Stem Cells-Origins and Biomarkers: Perspectives for Targeted Personalized Therapies. Front. Immunol. 11, 1280. 10.3389/fimmu.2020.01280 32849491 PMC7426526

[B92] Wang C. Jin H. Gao D. Lieftink C. Evers B. Jin G. (2018). Phospho-ERK Is a Biomarker of Response to a Synthetic Lethal Drug Combination of Sorafenib and MEK Inhibition in Liver Cancer. J. Hepatol. 69, 1057–1065. 10.1016/j.jhep.2018.07.004 30030148

[B93] Wang Q. Mu H. Zhang L. Dong D. Zhang W. Duan J. (2015). Characterization of Two Water-Soluble Lignin Metabolites with Antiproliferative Activities from Inonotus Obliquus. Int. J. Biol. Macromolecules 74, 507–514. 10.1016/j.ijbiomac.2014.12.044 25583019

[B94] Xu W. Liu K. Chen M. Sun J. Y. McCaughan G. W. Lu X. J. (2019). Immunotherapy for Hepatocellular Carcinoma: Recent Advances and Future Perspectives. Ther. Adv. Med. Oncol. 11, 1758835919862692. 10.1177/1758835919862692 31384311 PMC6651675

[B95] Yang S. Meng J. Yang Y. Liu H. Wang C. Liu J. (2016). A HSP60-Targeting Peptide for Cell Apoptosis Imaging. Oncogenesis 5, e201. 10.1038/oncsis.2016.14 26926787 PMC5154354

[B96] Zhang K. Wang T. Zhou H. Feng B. Chen Y. Zhi Y. (2018). A Novel Aurora-A Inhibitor (MLN8237) Synergistically Enhances the Antitumor Activity of Sorafenib in Hepatocellular Carcinoma. Mol. Ther. - Nucleic Acids 13, 176–188. 10.1016/j.omtn.2018.08.014 30292139 PMC6172479

[B97] Zhen X. Choi H. S. Kim J.-H. Kim S.-L. Liu R. Yun B.-S. (2020). Machilin D, a Lignin Derived from Saururus Chinensis, Suppresses Breast Cancer Stem Cells and Inhibits NF-Κb Signaling. Biomolecules 10, 245. 10.3390/biom10020245 PMC7072518 32033472

[B98] Zhou H. Du F. Li X. Zhang B. Li W. Yan B. (2008). Characterization of Organic Molecules Attached to Gold Nanoparticle Surface Using High Resolution Magic Angle Spinning 1H NMR. J. Phys. Chem. C 112, 19360–19366. 10.1021/jp806907c

[B99] Zhou J. Xiao H. Yang X. Tian H. Xu Z. Zhong Y. (2018). Long Noncoding RNA CASC9.5 Promotes the Proliferation and Metastasis of Lung Adenocarcinoma. Sci. Rep. 8, 37. 10.1038/s41598-017-18280-3 29311567 PMC5758512

