# Supplementary figures and images for: High-Molecular-Weight Fractions of Spruce and Eucalyptus Lignin as a Perspective Nanoparticle-Based Platform for a Therapy Delivery in Liver Cancer
Source: Front Bioeng Biotechnol. 2022 Feb 7;9:817768. doi: 10.3389/fbioe.2021.817768 (PMC8860172; doi:10.3389/fbioe.2021.817768)

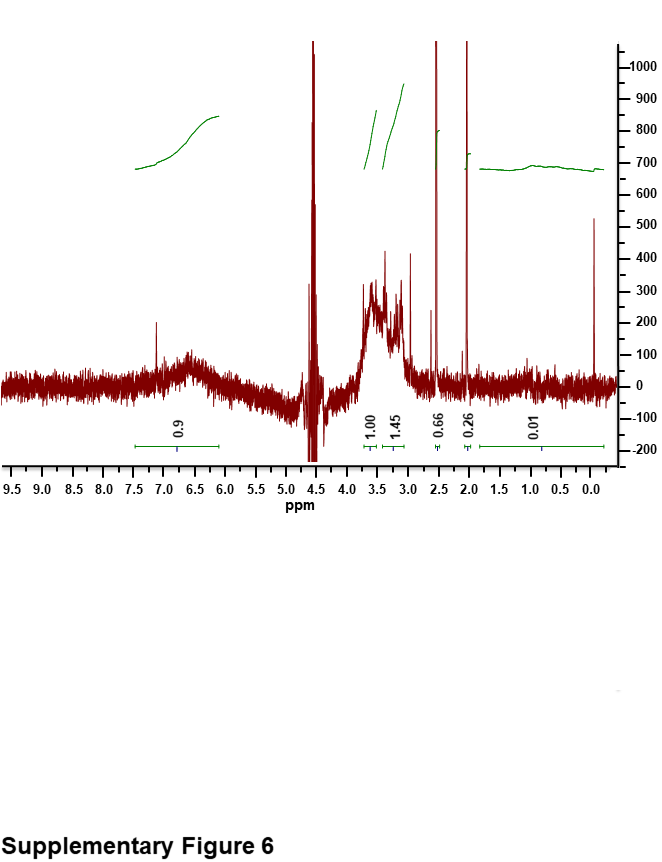

Supplement: Supplementary file 2 [file Image6.tif]

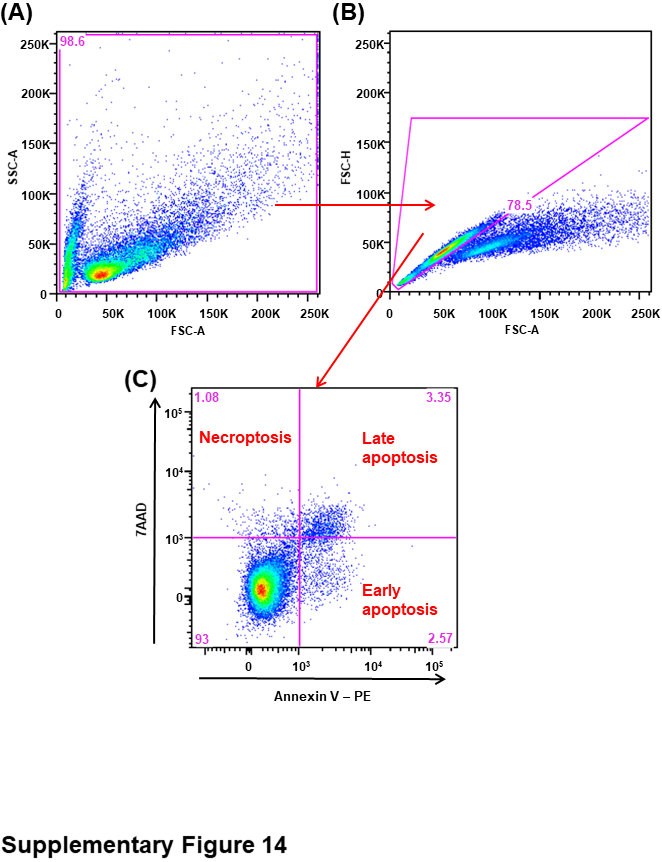

Supplement: Supplementary file 4 [file Image14.tif]

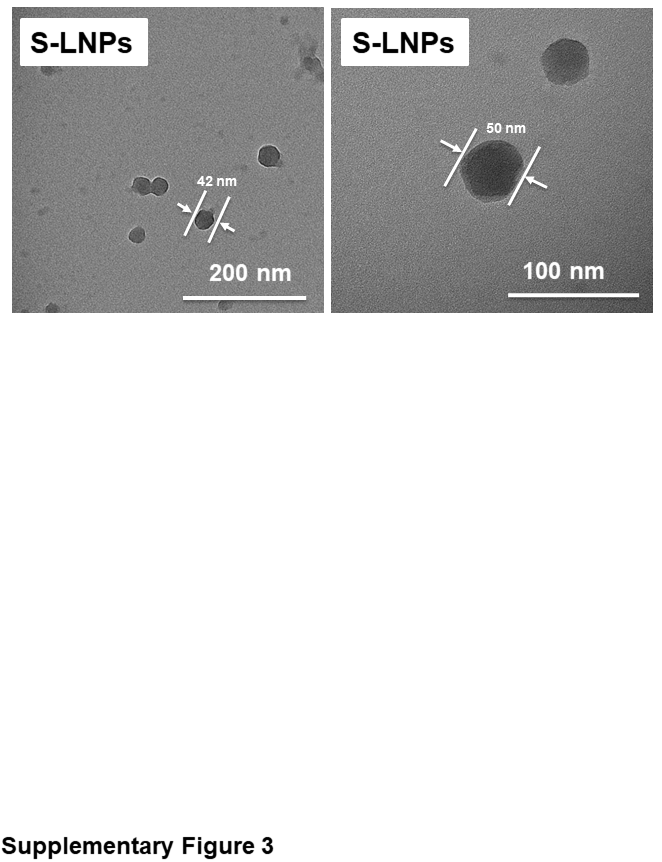

Supplement: Supplementary file 5 [file Image3.tif]

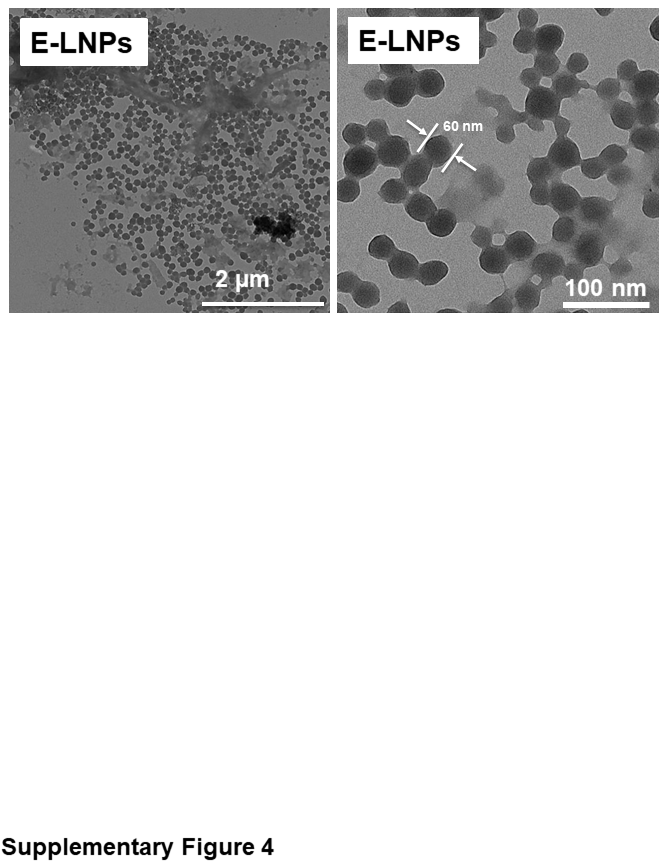

Supplement: Supplementary file 6 [file Image4.tif]

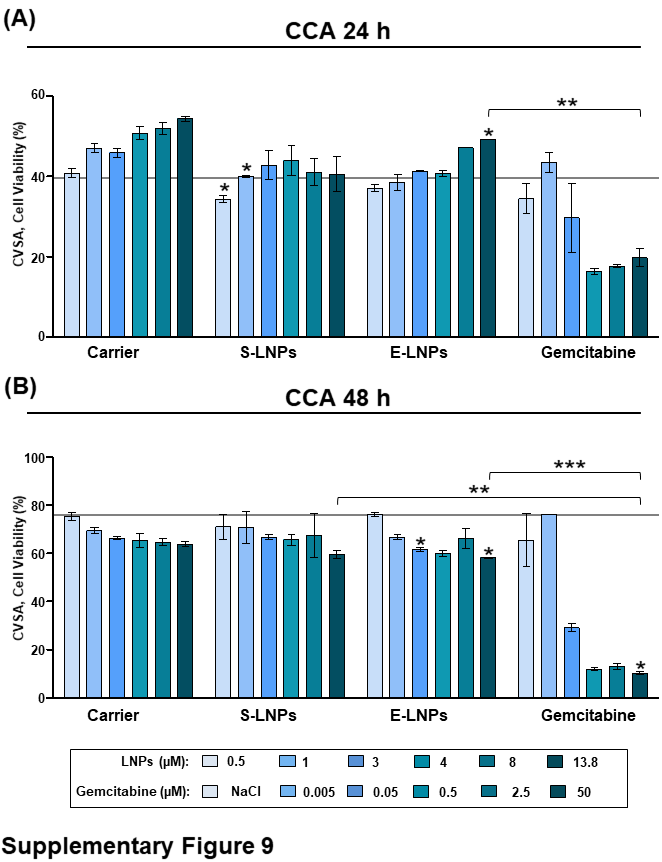

Supplement: Supplementary file 7 [file Image9.tif]

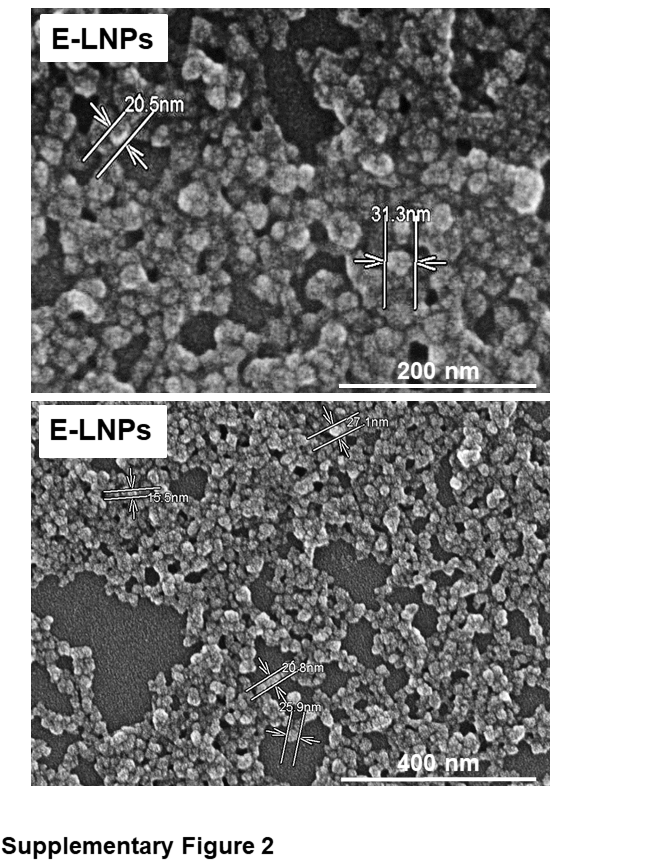

Supplement: Supplementary file 8 [file Image2.tif]

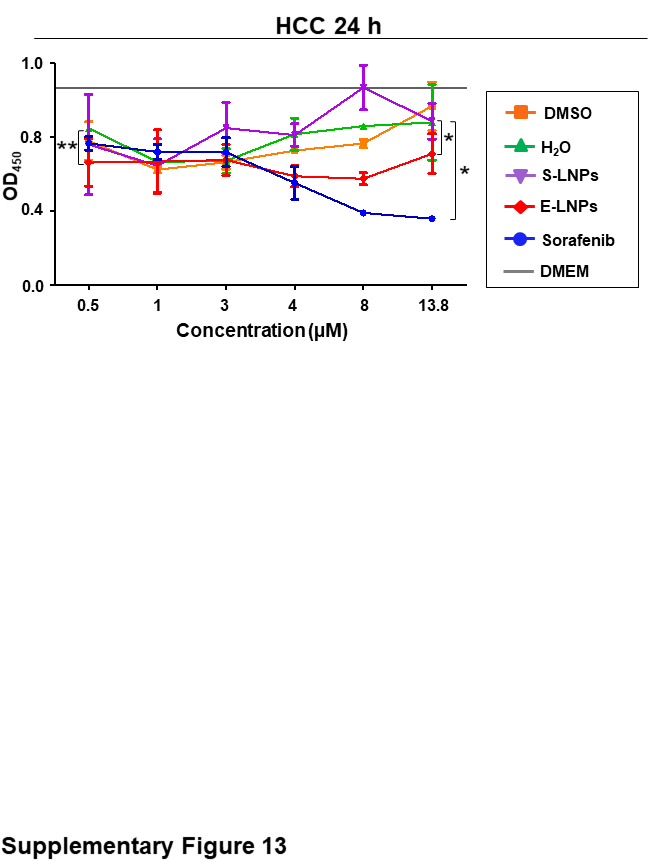

Supplement: Supplementary file 9 [file Image13.tif]

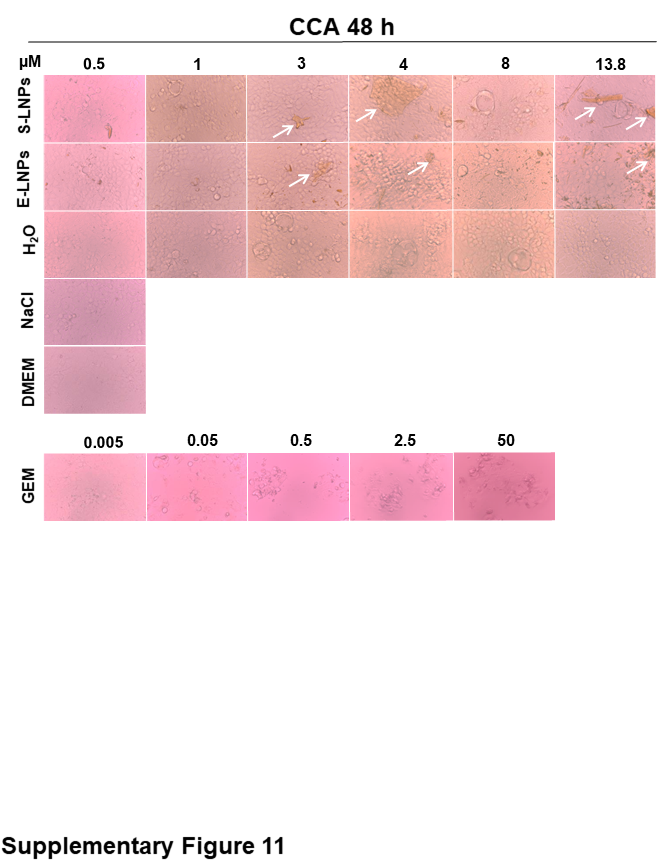

Supplement: Supplementary file 10 [file Image11.tif]

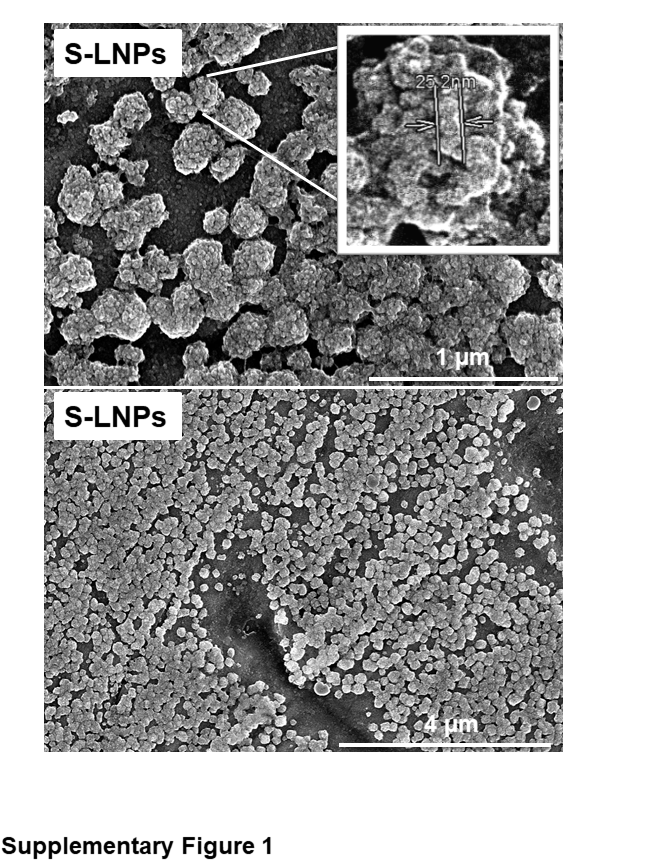

Supplement: Supplementary file 11 [file Image1.tif]

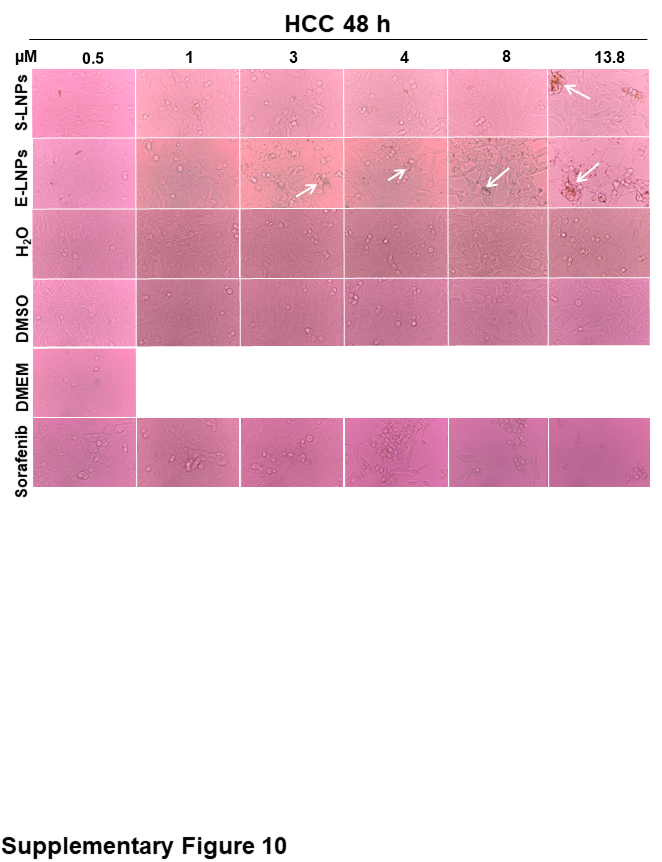

Supplement: Supplementary file 12 [file Image10.tif]

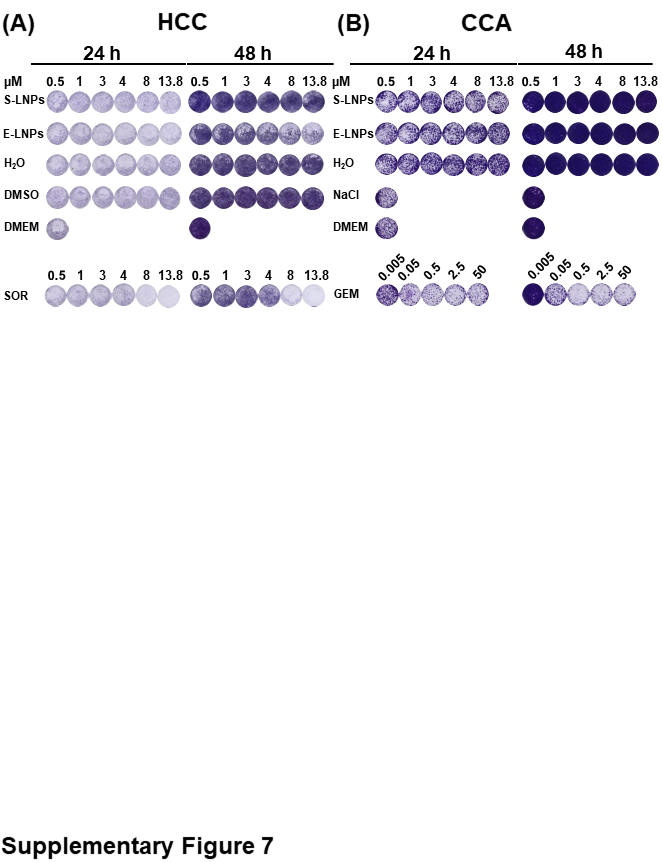

Supplement: Supplementary file 13 [file Image7.tif]

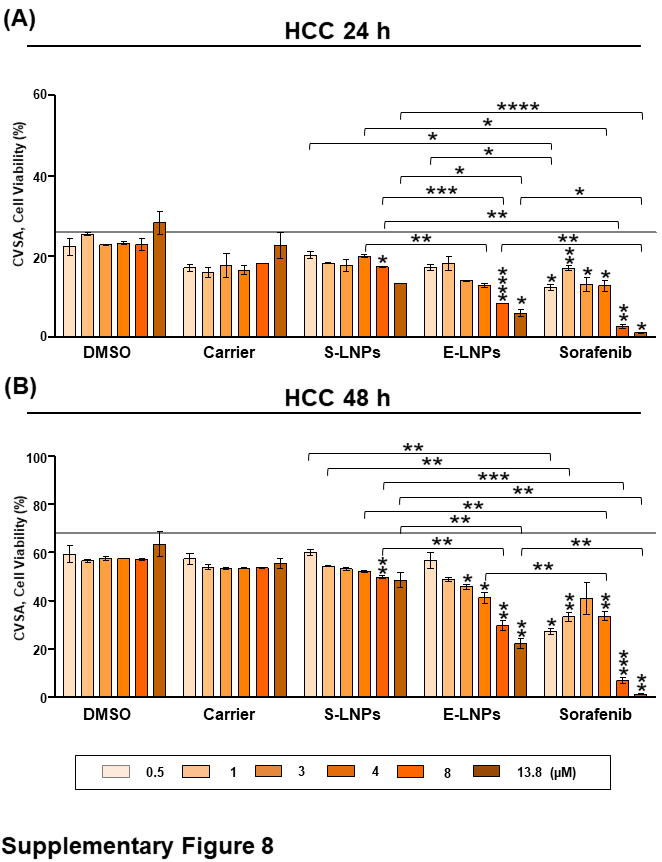

Supplement: Supplementary file 14 [file Image8.tif]

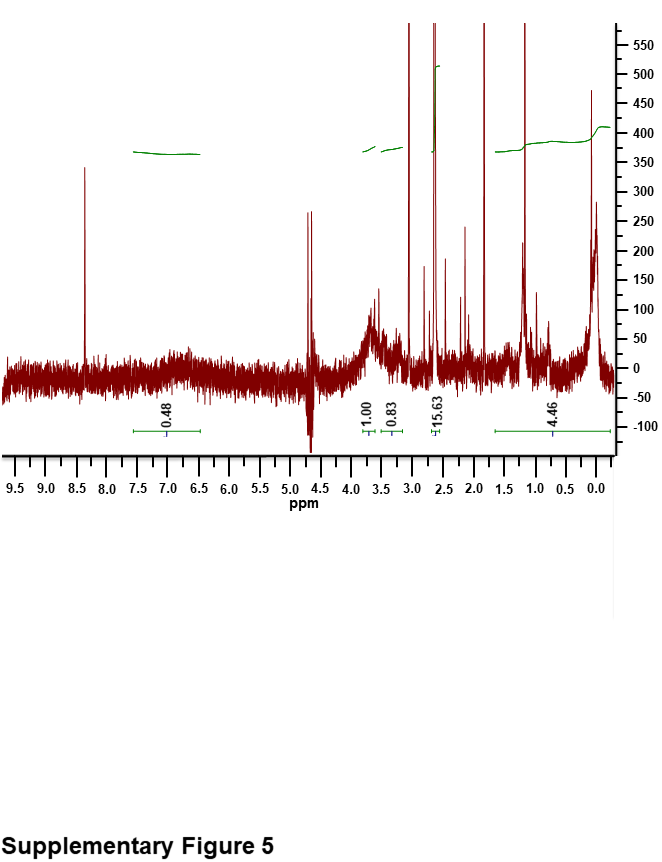

Supplement: Supplementary file 15 [file Image5.tif]

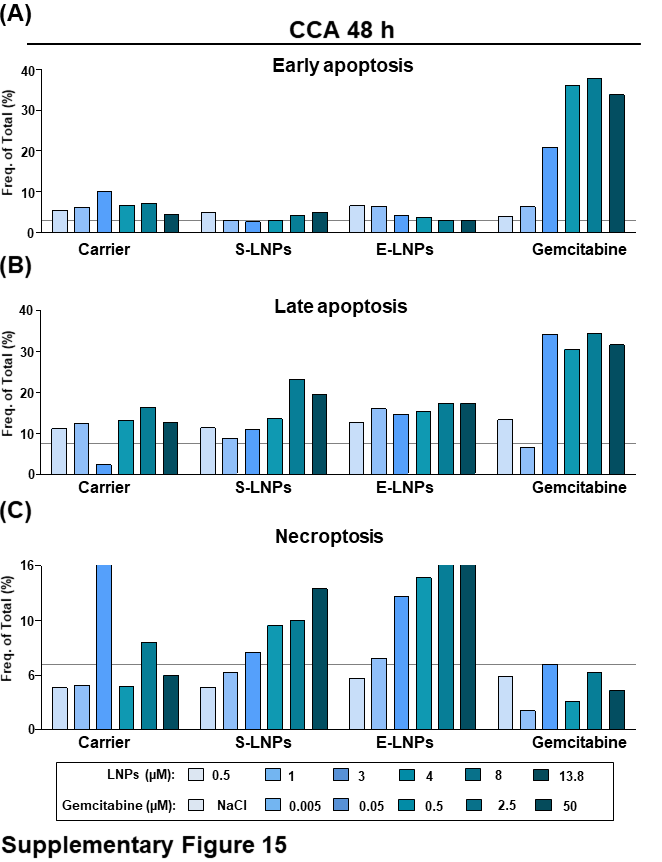

Supplement: Supplementary file 16 [file Image15.tif]

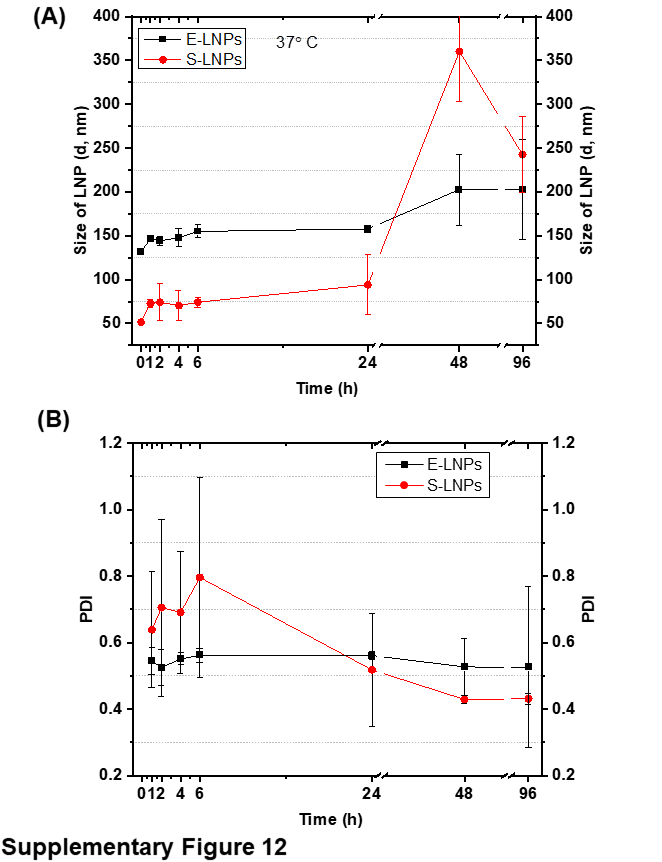

Supplement: Supplementary file 17 [file Image12.tif]
