# Supplementary material for: High-Molecular-Weight Fractions of Spruce and Eucalyptus Lignin as a Perspective Nanoparticle-Based Platform for a Therapy Delivery in Liver Cancer
Source: Front Bioeng Biotechnol. 2022 Feb 7;9:817768. doi: 10.3389/fbioe.2021.817768 (PMC8860172; doi:10.3389/fbioe.2021.817768)
Supplement: Supplementary file 3 [file Table1.pdf]

**LNPs**	**µg/ml**	0.03	0.06	0.19	0.21	0.50	0.86
	**Counts/ml**	4.77×10^{11}	9.55×10^{11}	2.86×10^{12}	3.29×10^{12}	7.64×10^{12}	1.32×10^{13}
**Standard therapy**	**Sorafenib (µM)**	0.5	1	3	4	8	13.8
	**Gemcitabine (µM)**	-	0.005	0.05	0.5	2.5	50

Supplementary Table 1
