# Supplementary material for: High-Molecular-Weight Fractions of Spruce and Eucalyptus Lignin as a Perspective Nanoparticle-Based Platform for a Therapy Delivery in Liver Cancer
Source: Front Bioeng Biotechnol. 2022 Feb 7;9:817768. doi: 10.3389/fbioe.2021.817768 (PMC8860172; doi:10.3389/fbioe.2021.817768)
Supplement: Supplementary file 18 [file DataSheet1.docx]

Supplementary Material

**List of Abbreviations**

**Aliph-OH** Aliphatic hydroxyls

**Annexin V-PE** Annexin V-phycoerythrin

**7-AAD** 7-amino-actinomycin D

**BBI** Double resonance broadband probe

**CaCl_2_** Calcium chloride

**CCA** Cholangiocarcinoma

**CCK-8** Cell counting kit – 8

**CDCl_3_**  Deuterochloroform

**CLPs** Colloidal lignin particles

**CO_2_** Carbon dioxide

**CPA** Cell proliferation assay

**CT** Computed tomography

**CVSA** Crystal violet staining assay

**DEPT** Distortionless enhancement by polarization transfer

**DLS** Dynamic light scattering

**DMEM** Dulbecco’s Modified Eagle Medium

**DMF** Dimethylformamid

**DMSO** Dimethyl sulfoxide

**D_2_O** Deuterium water

**e-HNDI** N-hydroxy-5-norbornene-2,3-dicarboxylic acid imide

**EtOAc** Ethyl acetate

**EtOH** Ethanol

**E-LNPs** Eucalyptus lignin nanoparticles

**S-LNPs** Spruce lignin nanoparticles

**FACS** Fluorescence-activated cell sorting

**FBS** Fetal bovine serum

**Fe_3_O_4_** iron oxide

**GC** Glycol chitosan

**HBV** Hepatitis B virus

**HCC** Hepatocellular carcinoma

**HCV** Hepatitis C virus

**HEPES** 4 - (2-hydroxyethyl) - 1- piperazineethanesulfonic acid

**HuCCT-1** Human cholangiocellular carcinoma

**HPLC** High-performance liquid chromatography

**HSQC** Heteronuclear single quantum correlation

**kDA** Kilodalton

**LCCs** Lignin-carbohydrate complexes

**LNPs** Lignin nanoparticles

**MeOH** Methanol

**MEM NEAA** Minimum essential medium non-essential amino acids

**MRI** Magnetic resonance imaging

**M*_w_*** Molecular weight

**NA** Not applicable

**N_2_** Nitrogen

**NaCl** Sodium chloride

**NIR** Near-infrared

**NMR** Nuclear magnetic resonance

**NPs** Nanoparticles

**ns** Not significant

***P*** *P values*

**OD** Optical density

**PDI** Polydispersity index

**PET** Positron emission tomography

**Ph-OH** Phenolic hydroxyls

**Pt-Pd** Platinum-palladium alloy

**Py-GC/MS/FID** Pyrolysis-gas/chromatography/mass spectrometry/flame ionization detector

**SEC** Size-exclusion chromatography

**SEM** Scanning electron microscopy

**S.E.M.** Standard error of the mean

**S-LNPs** Spruce nanoparticles

**TEM** Transmission electron microscopy

**THF** Tetrahydrofuran

**UN** United Nations

**UV** Ultraviolet

**Supplementary Tables**

**Supplementary Table 1 |** The concentration of LNPs and standard therapeutics used in this study.

**Supplementary Table 2 |** *P* values from the CVSA cell viability analysis comparing S- and E-LNPs treatment groups versus carrier and standard therapeutic agents in HCC and CCA.

**Supplementary Figure Legends**

**Supplementary Figure 1 |** The SEM micrographs of S-LNPs showing the formation of larger supra particle aggregates. Shown are SEM micrographs taken at different magnifications. Arrows indicate the particle size of approx. 25 nm.

**Supplementary Figure 2 |** The SEM micrographs of E-LNPs showing the formation of the surface layer of interconnected particles. Shown are SEM micrographs taken at different magnifications. Arrows indicate the particle size of approx. 15-30 nm. E-LNPs tend to form more interconnections and being deposited onto a hydrophilic silicon substrate, forming a relatively flat layer, as compared to S-LNPs **(**shown in **Supplementary Figure 1)**.

**Supplementary Figure 3 |** TEM micrographs of S-LNPs, showing the average size of 40-50 nm. The particles were synthesized at the initial lignin concentration of 1 mg/ml.

**Supplementary Figure 4 |** TEM micrographs of E-LNPs showing a large number of interconnections between E-LNPs. The particles were synthesized at the initial lignin concentration of 1 mg/ml.

**Supplementary Figure 5 |** Integrated NMR spectra of S-LNPs demonstrating the presence of more intense signal for nonpolar aliphatic side chains of lignin (0.0-2.0 ppm). Shown are NMR spectra of aqueous suspensions of S-LNPs. The particles were synthesized at the initial lignin concentration of 1 mg/ml and then mixed with D_2_O. The spectra were integrated so that the intensity of the methoxy group (3.8-3.6 ppm) was 1.0.

**Supplementary Figure 6 |** Integrated NMR spectra of E-LNPs demonstrating the presence of higher numbers of carbohydrates on their surfaces. Shown are NMR spectra of aqueous suspensions of E-LNPs which revealed carbohydrates- and aromatic units-rich regions. The particles were synthesized at the initial lignin concentration of 1 mg/ml and then mixed with D_2_O. The spectra were integrated so that the intensity of the methoxy group (3.8-3.6 ppm) was 1.0.

**Supplementary Figure 7 |** LNPs possessed inhibitory capacity towards HCC cell line, whereas no inhibition was observed towards CCA cell line. **(A)** HCC and **(B)** CCA cell lines were treated with two types of LNPs (S-LNPs or E-LNPs), as well as standard therapeutics, sorafenib and gemcitabine, respectively. The inhibitory capacity of LNPs was assessed using CVSA at two time points 24 h and 48 h post-incubation. Carriers (DMSO for sorafenib, NaCl for gemcitabine and H_2_O for LNPs) were used as negative controls. DMEM represents an untreated group. Both standard therapeutics were also given at their plasma concentration (13.8 µM for sorafenib ([Fucile et al., 2015](#_ENREF_1)) and 50 µM for gemcitabine ([Fujiwara et al., 2015](#_ENREF_2))).

**Supplementary Figure 8 |** LNPs possessed inhibitory capacity towards HCC cell line as confirmed by CVSA analysis using ImageJ. Cell viability analysis performed **(A)** 24 h and **(B)** 48 h post-incubation. Values represent mean ± standard error of the mean (S.E.M). Significance levels were depicted as: **P* < 0.05, ***P* < 0.01, ****P* < 0.001 and *****P* < 0.0001. Detailed *P* values for all groups are displayed in **Supplementary Table 2**.

**Supplementary Figure 9 |** LNPs possessed no inhibitory capacity towards CCA cell line as confirmed by CVSA analysis using ImageJ. Cell viability analysis performed **(A)** 24 h and **(B)** 48 h post-incubation. Values represent mean ± S.E.M. Significance levels were depicted as: **P* < 0.05, ***P* < 0.01, ****P* < 0.001 and *****P* < 0.0001. Detailed *P* values for all groups are displayed in **Supplementary Table 2**.

**Supplementary Figure 10 |** Microscopy showed a dose-dependent inhibition of HCC cells and a dose-dependent accumulation of yellowish conglomerates in LNPs-treated groups. HCC cells were treated with different concentrations of S-LNPs, E-LNPs and the standard therapy sorafenib. Carriers (DMSO for sorafenib and H_2_O for LNPs) were used as negative controls, DMEM represents an untreated group. Bright-field microscopy (magnification 40x) was performed at 48 h post-incubation. Yellowish conglomerates are depicted with the white arrows.

**Supplementary Figure 11 |** Despite the development of yellowish conglomerates, no inhibitory effect of LNPs was observed in CCA cells. CCA cells were treated with different concentrations of S-LNPs, E-LNPs and the standard therapy gemcitabine. Carriers (NaCl for gemcitabine and H_2_O for LNPs) were used as negative controls, DMEM represents an untreated group. Bright-field microscopy (magnification 40x) was performed at 48 h post-incubation. Yellowish conglomerates are depicted with the white arrows. Standard therapy gemcitabine was also given at plasma concentration 50 µM ([Fujiwara et al., 2015](#_ENREF_2)).

**Supplementary Figure 12 |** Heating of E-LNPs and S-LNPs at 37°C led to increased particle size. The LNPs synthesized at the initial lignin concentration of 1 mg/ml were heated at 37°C for 96 h, to track the size change. The average diameter of E-LNPs at 37°C was gradually increasing over time, namely from 132 nm in the beginning to 202 nm after 96 h. **(A)** Shown is the average diameter of S-LNPs that increased from 51 nm to 93 nm after 24 h at 37°C, and increased to 242 nm after 96 h. **(B)** Shown is PDI, which is slightly decreasing for S-LNPs upon heating overtime, whereas PDI of E-LNPs remained constant.

**Supplementary Figure 13 |** LNPs demonstrated inhibitory effect on HCC cell line in CCK-8 analysis. HCC cells were treated with LNPs (S-LNPs and E-LNPs) as well as the standard therapeutic sorafenib and 24 h post-incubation cells were subjected to CCK-8 analysis. Optical density (OD_450_) was detected spectrophotometrically using a microplate reader Infinite 200 PRO Nano Quant Tecan. Shown are mean ± S.E.M., with **P* < 0.05, ***P* < 0.01.

**Supplementary Figure 14 |** Gating strategy for FACS analysis to measure early- and late apoptosis, as well as necroptosis. FACS analysis using Annexin V-PE and 7-AAD staining was performed using both HCC and CCA cell lines after the treatment with LNPs and standard therapeutics. **(A)** Cells were gated using forward- and side scatter characteristics and **(B)** duplicates were outgated. **(C)** Thereafter analysis of three different populations based on 7-AAD and Annexin V-PE staining was performed. The upper left quadrant indicates necroptotic, the lower right and upper right quadrants indicate early- and late apoptotic cells, respectively.

**Supplementary Figure 15 |** FACS analysis performed 48 h after the incubation induced early- and late apoptosis in gemcitabine-treated groups and mostly necroptosis in S-LNPs- and E-LNPs-treated groups. CCA cells were treated with S-LNPs and E-LNPs fractions as well as the standard therapeutic gemcitabine. FACS analysis to detect **(A)** early- and **(B)** late apoptosis, as well as **(C)** necroptosis was performed. A positive control (gemcitabine) was applied at different concentrations increasing from 0.005 until 50 µM. NaCl was used as a negative control for gemcitabine group and a carrier (H_2_O) was used for LNPs. Shown are frequencies of **(A)** early apoptotic, **(B)** late apoptotic and **(C)** necroptotic cells in percent. The grey line represents the values for the control group DMEM.

**Supplementary References**

Fucile, C., Marenco, S., Bazzica, M., Zuccoli, M.L., Lantieri, F., Robbiano, L., Marini, V., Di Gion, P., Pieri, G., Stura, P.*, et al.* (2015). Measurement of sorafenib plasma concentration by high-performance liquid chromatography in patients with advanced hepatocellular carcinoma: is it useful the application in clinical practice? A pilot study. Medical oncology (Northwood, London, England) *32*, 335.

Fujiwara, Y., Kobayashi, S., Nagano, H., Kanai, M., Hatano, E., Toyoda, M., Ajiki, T., Takashima, Y., Yoshimura, K., Hamada, A.*, et al.* (2015). Pharmacokinetic Study of Adjuvant Gemcitabine Therapy for Biliary Tract Cancer following Major Hepatectomy (KHBO1101). PLOS ONE *10*, e0143072.
